# Lipid Dependence of Xanthophyll Cycling in Higher Plants and Algae

**DOI:** 10.3389/fpls.2020.00455

**Published:** 2020-04-21

**Authors:** Reimund Goss, Dariusz Latowski

**Affiliations:** ^1^Department of Plant Physiology, Institute of Biology, Leipzig University, Leipzig, Germany; ^2^Department of Plant Physiology and Biochemistry, Faculty of Biochemistry, Biophysics and Biotechnology, Jagiellonian University, Kraków, Poland

**Keywords:** fatty acid, lipid, MGDG, thylakoid membrane domain, violaxanthin de-epoxidase, xanthophyll cycle

## Abstract

The xanthophyll cycles of higher plants and algae represent an important photoprotection mechanism. Two main xanthophyll cycles are known, the violaxanthin cycle of higher plants, green and brown algae and the diadinoxanthin cycle of Bacillariophyceae, Xanthophyceae, Haptophyceae, and Dinophyceae. The forward reaction of the xanthophyll cycles consists of the enzymatic de-epoxidation of violaxanthin to antheraxanthin and zeaxanthin or diadinoxanthin to diatoxanthin during periods of high light illumination. It is catalyzed by the enzymes violaxanthin or diadinoxanthin de-epoxidase. During low light or darkness the back reaction of the cycle, which is catalyzed by the enzymes zeaxanthin or diatoxanthin epoxidase, restores the epoxidized xanthophylls by a re-introduction of the epoxy groups. The de-epoxidation reaction takes place in the lipid phase of the thylakoid membrane and thus, depends on the nature, three dimensional structure and function of the thylakoid lipids. As the xanthophyll cycle pigments are usually associated with the photosynthetic light-harvesting proteins, structural re-arrangements of the proteins and changes in the protein-lipid interactions play an additional role for the operation of the xanthophyll cycles. In the present review we give an introduction to the lipid and fatty acid composition of thylakoid membranes of higher plants and algae. We introduce the readers to the reaction sequences, enzymes and function of the different xanthophyll cycles. The main focus of the review lies on the lipid dependence of xanthophyll cycling. We summarize the current knowledge about the role of lipids in the solubilization of xanthophyll cycle pigments. We address the importance of the three-dimensional lipid structures for the enzymatic xanthophyll conversion, with a special focus on non-bilayer lipid phases which are formed by the main thylakoid membrane lipid monogalactosyldiacylglycerol. We additionally describe how lipids and light-harvesting complexes interact in the thylakoid membrane and how these interactions can affect the structure of the thylakoids. In a dedicated chapter we offer a short overview of current membrane models, including the concept of membrane domains. We then use these concepts to present a model of the operative xanthophyll cycle as a transient thylakoid membrane domain which is formed during high light illumination of plants or algal cells.

## Introduction

Xanthophyll cycles, which consist of the de-epoxidation of epoxy-xanthophylls during high light and the epoxidation of epoxy-free xanthophylls during low light or darkness, are found in all eukaryotic photoautotrophs (for reviews see Goss and Jakob, 2010; Niyogi and Truong, 2013; Goss and Lepetit, 2015). The xanthophyll cycles act as an important protection mechanism against damage of the photosynthetic apparatus by supersaturating light conditions (for reviews see Horton, 2014; Ruban, 2016). The main part of photoprotection provided by the xanthophyll cycles operates on the time-scale of minutes and thus allows the plants to react to short-term changes of the light intensities in their natural environment (Demmig-Adams and Adams, 2006; Demmig-Adams et al., 2014). For higher plants fast fluctuations of the light intensity can be induced by clouds or by rapid changes of the leaf coverage in shaded environments such as the tropical rainforests. For algae even moderate water mixing can result in a rapid change of the light intensity from full sunlight to almost complete darkness. In addition, tidal changes affect the light exposure of those species inhabiting the coastal regions. Besides the fast photoprotection the xanthophyll cycles provide long-term protection which lasts for days, weeks or even months (Demmig-Adams and Adams, 2006; Demmig-Adams et al., 2014). These long-lasting photoprotection components can be observed in evergreen plant species which are exposed to prolonged environmental stress like the combination of cold temperatures and high light intensities during the winter months.

The observation that changes in the light intensities lead to inter-conversions of specific leaf xanthophylls dates back to the late 1950s/early 1960s (Hager, 1957; Saphozhnikov et al., 1957; Yamamoto et al., 1962). These first experiments revealed that the xanthophyll violaxanthin (Vx) is converted to the intermediate xanthophyll antheraxanthin (Ax) and then finally to zeaxanthin (Zx) during high light illumination. Later, it was demonstrated that darkness or low light lead to the reversal of the light-driven xanthophyll inter-conversions (Hager, 1967a) and the term Vx cycle was introduced (section Types of Xanthophyll Cycles). In addition, the existence of a second xanthophyll cycle, namely the diadinoxanthin (Ddx) cycle, in several groups of algae was reported (Hager and Stransky, 1970; Stransky and Hager, 1970). Newer measurements have shown that, besides the dominant Vx- and Ddx cycles, further less common light-driven cyclic inter-conversions of xanthophylls exist (Rabinowitch et al., 1975; Goss et al., 1998; Bungard et al., 1999). The following investigations were concerned with the characteristics of the enzymes which carry out the de-epoxidation and epoxidation reactions such as pH-optimum and co-substrate requirements (Hager, 1967a, b; section Reaction Sequences and Xanthophyll Cycle Enzymes). With regard to the function of the xanthophyll cycles it took until the late 1980s that a connection between the conversion of Vx to Zx and the quenching of chlorophyll a fluorescence, which indicates a thermal dissipation of excitation energy, could be obtained and described (Demmig et al., 1987, 1988, section Function of Xanthophyll Cycles). Since then numerous studies have dealt with this process which was called NPQ (reviewed in Horton, 2014; Ruban, 2016) and even today work on the molecular mechanism of NPQ in higher plants and algae represents an important research topic. With respect to the localization of the xanthophyll cycle pigments it was clear from the beginning that the pigments are located within the chloroplast. Later studies have presented evidence that within the plastidic thylakoid membranes the xanthophyll cycle pigments are associated with the light-harvesting complexes of the photosystems (Bassi et al., 1993; Ruban et al., 1994, section Localization of Xanthophyll Cycle Pigments in the Thylakoid Membrane). Regarding the main topic of the present review, i.e., the influence of lipids on the operation of the xanthophyll cycles, first evidence that the main thylakoid membrane monogalactosyldiacylglycerol (MGDG) plays an important role in the conversion of Vx to Ax and Zx dates back to the 1970s (Yamamoto and Higashi, 1978, sections Lipid Classes and Lipids as Solvents for Xanthophyll Cycle Pigments). Later measurements demonstrated that the thylakoid membrane lipids, and especially MGDG, act as solvents of the xanthophyll cycle pigments (Latowski et al., 2004; Goss et al., 2005, section Lipids as Solvents for Xanthophyll Cycle Pigments) and that special three-dimensional lipid structures or phases, i.e., non-bilayer lipid phases, are needed for the efficient operation of the xanthophyll cycles of higher plants and algae (Latowski et al., 2002, 2004; Goss et al., 2005, 2007, sections Three Dimensional Structures of Lipids and Role of Non-bilayer Lipid Phases for Xanthophyll Cycling). Additional investigations could show that the xanthophyll cycle pigments are not only associated with the light-harvesting proteins via special protein binding sites but also exist in lipid shields surrounding the complexes (Lepetit et al., 2010; Schaller et al., 2010; section Localization of Non-bilayer Lipid Phases in the Thylakoid Membrane). Recent research on the lipid dependence of xanthophyll cycling has focused on the localization of the non-bilayer lipid phases within the thylakoid membrane (Garab et al., 2017, section Localization of Non-bilayer Phases Involved in Xanthophyll Cycling) and how these structures are formed during high light illumination (Goss et al., 2007; Jahns et al., 2009, section Formation of Non-bilayer Lipid Phases by Structural Changes of Light-Harvesting Proteins). Models describing the operation of the xanthophyll cycle within the thylakoid membrane have also included information from the newest concepts on the structure and function of biological membranes (Goni, 2014; Nicholson, 2014, section The Xanthophyll Cycle Membrane Domain in the Light of Recent Membrane Models).

## Lipid Composition of Thylakoid Membranes of Higher Plants and Algae

### Lipid Classes

One of the most important biological functions of lipids is the formation of membranes within the cell and the cell organelles. In plant cells the largest membrane system is the thylakoid membrane within the chloroplast. Thylakoid membranes, like the inner membranes of mitochondria, are characterized by a high protein per lipid ratio which already indicates that the thylakoid lipids play an important role not only as membrane building blocks, but also as regulators of the structure and function of both integral and peripheral membrane proteins, including the photosynthetic complexes or the xanthophyll cycle enzymes (Krauss, 2001; Kirchhoff et al., 2002; Kobayashi et al., 2016, see also section Xanthophyll Cycles of Higher Plants and Algae). In the thylakoid membranes representatives of the following three lipid classes are present: glycosylglycerol lipids (GGLs), glycerophospholipids (GPs), and prenol lipids (PRs). Both GGL and GP molecules are characterized by the presence of two fatty acyl residues and one trihydric alcohol, namely glycerol. The main difference between these two lipid groups is the presence of a phosphate residue attached to glycerol in GPs, whereas in GGL molecules one or more sugar residues are directly associated with glycerol through glycosidic bonds (Li-Beisson et al., 2016). PRs also represent an important group of thylakoid lipids. They include, among others, quinones such as plastoquinone, phylloquinone (vitamin K_1_) or tocopherols, as well as isoprenoids such as the carotenoids or the phytol chains of the chlorophyll molecules (Li-Beisson et al., 2016). PRs, and especially carotenoids, play an important role for the variability of the thylakoid lipid composition and can be used for the classification of various groups of algae.

**FIGURE 1:**
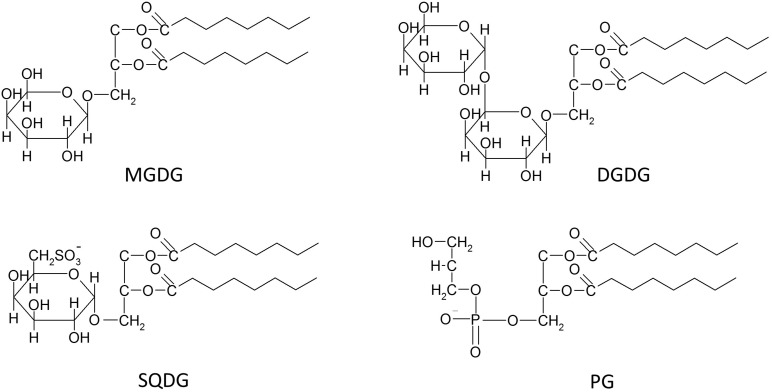
Schematic structures of the four main thylakoid membrane lipids, i.e., the three glycosylglycerol lipids (GGLs) monogalactosyldiacylglycerol (MGDG), digalactosyldiacylglycerol (DGDG) and sulfoquinovosyldiacylglycerol (SQDG), and the one glycerophospholipid (GP) phosphatidylglycerol (PG).

While the thylakoid membranes of different photoautotrophs may differ in the nature of their prenol lipids, the other lipid classes appear to be comparable for different types of thylakoids. Differences are not observed in the general structure of the different lipid classes but rather in their fatty acid (FA) composition and their contribution to the total thylakoid membrane lipid. The most common lipids of thylakoid membranes are three types of GGLs and one representative of the GPs (Figure 1). Among the lipids belonging to the GGLs two lipids, namely digalactosyldiacylglycerol (DGDG) and monogalactosyldiacylglycerol (MGDG), contain a galactopyranosyl residue, whereas the sulfolipid sulfoquinovosyldiacylglycerol (SQDG) carries a glucopyranosyl residue with a sulfonic group at the C6 position of the glucose residue. The first galactose residues of MGDG and DGDG are β-anomeric forms, while the second galactose of DGDG, as well as the glucose residue of SQDG are α-sugars (Hölzl and Dörmann, 2019). In addition to MGDG, DGDG, and SQDG phosphatidylglycerol (PG), a lipid belonging to the class of GPs, is present in the thylakoid membrane (Harwood and Guschina, 2009; Figure 1). While DGDG and MGDG are neutral lipids, SQDG and PG are negatively charged due to the presence of the sulfate and phosphate residues, respectively (Boudiere et al., 2014). The presence of three other representatives of the GPs, namely phosphatidylinositol (PI), phosphatidylcholine (PC), and in the case of the green alga *Chlamydomonas reinhardii* phosphatidylethanolamine (PE), in thylakoid membranes has been proposed. However, it is still under discussion if these lipids really represent specific components of the inner thylakoid membrane. While in all thylakoids isolated from both plants and algae PI was commonly detected with concentrations up to 5% of the total membrane lipid, it is not clear whether PC and PE simply represent contaminations of thylakoid preparations with lipids from membranes rich in GPs, such as cellular or mitochondrial membranes. In addition, nothing is known so far about the putative role of these three GPs in photosynthesis (Boudiere et al., 2014).

Whereas the presence of PI, and especially that of PC or PE, in thylakoid membranes still needs verification, MGDG, DGDG, SQDG, and PG unequivocally represent the typical lipids of all oxygenic photosynthetic membranes, ranging from cyanobacteria to thylakoids of algae and plants (Boudiere et al., 2014; Kobayashi et al., 2016). Among these four thylakoid lipids, MGDG contributes up to 50% to the total higher plant membrane lipid, DGDG contributes approximately 25%, whereas the content of PG reaches up to 10% and SQDG oscillates in the range from about 2 to 20%. Despite the fact that SQDG is widely considered as the least concentrated of the four main thylakoid lipids, many results have shown that especially the SQDG levels are highly variable in response to different environmental conditions. Thylakoid membranes of higher plants or marine cyanobacteria under phosphate depletion can show maximum levels of SQDG of up to 50 or even 70%, respectively. Additionally, under conditions of phosphate limitation, PG seems to be the only representative of the GP lipids with a content of solely 2% of the total membrane lipid of marine cyanobacteria (van Mooy et al., 2006; Shimojima, 2011; Boudiere et al., 2014). A replacement of PG by SQDG is commonly observed and is considered to be a ubiquitous phenomenon in photosynthetic organisms. Keeping constant the concentrations of the negatively charged lipids most likely ensures the stability of an anionic lipid environment within the photosynthetic membranes under phosphate limitation (Boudiere et al., 2014). With respect to function, SQDG appears to be important for the protection of PSII in halophytes. In general, the level of SQDG was found to be considerably higher in many salt treated halophytes, e.g., in *Aster tripolium*, *Sesuvium portulacastrum*, or *Crithmum maritimum*, although no changes in the sulfolipid contents were observed in glycophytes which were treated with different salt concentration (Ben Hamed et al., 2005; Ramani et al., 2006). In the light of these results it is possible that the high levels of SQDQ reported for various marine photoautotrophs represent an adaptation to the high salt concentration in their natural environment. In 2013 a small amount of a new negatively charged glycolipid was detected in several plant species under phosphorus depletion. The lipid was identified as glucuronosyldiacylglycerol (GlcADG), which contains a glucuronic acid instead of the glucose residue (Okazaki et al., 2013). GlcADG is synthesized by sulfoquinovosyltransferase SQD2, the same glycosyltransferase which is located in the inner envelope membrane of the chloroplast and is responsible for SQDG synthesis in plants and eukaryotic algae (Yu et al., 2002).

In general, the level of SQDG is significantly higher in cyanobacteria and algae than in plants. Within the algae diatoms, brown and red algae seem to be characterized by higher SQDG contents compared with the green algae. High concentrations of SQDG have been reported for the pennate diatom *Phaeodactylum tricornutum* and the centric diatoms *Cyclotella meneghiniana* and *Skeletonema* sp. (Goss et al., 2009; Yan et al., 2011; Lepetit et al., 2012). The high SQDG contents were especially obvious in thylakoid membranes isolated from cultures of *P. tricornutum* or *C. meneghiniana* which were grown under high light intensities (Lepetit et al., 2012). In these membranes SQDG represented the most abundant lipid and SQDG and PG contributed to more than 50% to the total membrane lipid. The ratio of neutral to negatively charged lipids was in the range between 1 and 2 whereas in higher plants and green algae values between 3 and 4 are typically observed. The data suggest a significantly higher negative charge of diatom thylakoid membranes compared to the thylakoids of higher plants or green algae. The significance of these extremely charged membranes for the diatom physiology is, however, still unknown (Vieler et al., 2007; Goss and Wilhelm, 2009).

Whereas SQDG and MGDG are almost undetectable in extraplastidic membranes of algae and plant cells, DGDG was shown to be able to substitute phospholipids in non-photosynthetic membranes, like the tonoplast (Andersson et al., 2005), the mitochondrial (Jouhet et al., 2004) or other plasma membranes (Andersson et al., 2003). Replacement of phospholipids by DGDG usually takes place under phosphate-deficient conditions (Härtel and Benning, 2000; Li et al., 2006). Under these limitations DGDG can amount to high concentrations and DGDG concentrations of up to 25% of the total membrane lipid were found in the plasma membranes and tonoplasts of oat root cells (Andersson et al., 2005). DGDG together with MGDG, seems to play a key role in the stabilization of chloroplast membranes under various kinds of environmental stresses such as drought, exposure to ozone, cold or heat stress, which generally result in an increase of the DGDG to MGDG ratio in the photosynthetic membrane (van Besouw and Wintermans, 1978; Heinz and Roughan, 1983; Heemskerk et al., 1986; Chen et al., 2006; Moellering et al., 2010; Moellering and Benning, 2011). This is not surprising, considering that MGDG is a substrate for DGDG synthases which belong to the glycosyltransferases and are upregulated by environmental stress like e.g., phosphate limitation. With respect to the role of MGDG, reduction of the MGDG concentrations of the thylakoid membrane by a down-regulation of MGDG synthases led to an impairment of the photosynthetic performance and photoautotrophic growth (Kobayashi et al., 2007). Moreover, in one of two MGDG-deficient mutants of *Arabidopsis thaliana*, *mgd1-1*, with about 40% less MGDG compared to the wild-type plant, a limitation of the Vx de-epoxidation to Ax and Zx was observed (see section Xanthophyll Cycles of Higher Plants and Algae). The reason for this limitation is an almost 40% reduction of the proton conductivity of the thylakoid membrane of the *mgd1-1* mutant under light stress (more than 1000 μmol photons m^–2^ s^–1^, Aronsson et al., 2008). The lower MGDG concentrations of the *mgd1-1* mutant thylakoid membrane led to an increased permeability of the membrane for protons and thus a decreased lumen acidification, resulting in a decreased pH-dependent activation of the xanthophyll cycle enzyme Vx de-epoxidase (VDE, see section Xanthophyll Cycles of Higher Plants and Algae).

Besides the lipids described above, two further lipids were identified in the thylakoid membranes of the green alga *C. reinhardtii*. One of them, acylsulfoquinovosyldiacylglycerol belongs to the GGLs whereas the other lipid, diacylgyceryl-N-trimethylhomoserine (DGTS), is a representative of the betaine lipids. DGTS was also detected in several other species of green algae as well as in ferns and mosses, but it is unknown if this lipid really represents a genuine thylakoid membrane lipid (Goss and Wilhelm, 2009). Similarly, other exotic lipids, which are often characteristic for only one algal species, such as the betaine lipids DGTA (diacylglycerylhydroxymethyl-N,N,N-trimethyl-β-alanine) and DGCC (diacylglycerylcarboxyhydroxymethylcholine) are found in brown algae or diatoms (Guschina and Harwood, 2006; Goss and Wilhelm, 2009).

However, in the case of *C. reinhardtii* it was not only demonstrated that DGTS represents a thylakoid lipid, but also that thylakoid DGTS is richer in trienes and C20 FAs than DGTS of other cellular membranes, which contains equal amounts of saturated and tetraene FAs (Janero and Barrnett, 1982). Interestingly, not only the composition of *C. reinhardtii* thylakoid membranes involves untypical lipids, but also the VDE of this algae seems to be unique (see also section Xanthophyll Cycles of Higher Plants and Algae). The enzyme is located at the stromal side of the thylakoid membrane and is related to a lycopene cyclase of photosynthetic bacteria but not to the typical VDE of plants or other algae (Li et al., 2016). Interestingly, the unique lipid and VDE composition of *C. reinhardtii* may be seen as one of the indicators of a strong relationship between the xanthophyll de-epoxidases and the lipid composition of the thylakoid membrane.

### Fatty Acids

Both the cross-species acclimation and the species-specific adaptation of biological membranes to various environmental conditions are based not only on changes of the stoichiometry of the individual lipid classes but also on changes of the respective FA residues of the lipids.

In plant cells, plastids, including chloroplasts, play the most important role in these adaptation processes since they represent the organelles where about 95% of the total plant FAs are produced (Ohlrogge et al., 1979). The fundamental importance of FAs was shown by null mutations in many single locus genes of FA synthesis which resulted in plant death during gamete or embryo development. Mutations in genes responsible for the later steps of the GP lipid synthesis seem to be not as deleterious for the establishment of photosynthetically active chloroplasts like mutations in the genes for FA synthesis (Hölzl and Dörmann, 2019).

The first products of phosphatidic acid (PA) acylation in chloroplasts are mainly PAs with residues of oleic acid (18:1) at the sn-1 and palmitic acid (16:0) at the sn-2 position, because the plastidic acyltransferases are specific for 16:0 and 18:1 acyl groups (Frentzen et al., 1983). Subsequently, after dephosphorylation of PA to diacylglycerol (DAG), FA specific desaturases (FADs) form double bonds in the acyl groups. This results in the transformation of 18:1 into linoleic (18:2) and α-linolenic (18:3) acid and the conversion of 16:0 into hexadecenoic (16:1) and hexadecatrienoic (16:3) FA residues. These FAs are commonly found in thylakoid PG (especially 16:1) and MGDG (especially 16:3), as a result of the so-called prokaryotic pathway of FA incorporation into glycerolipids (Higashi and Saito, 2019). On this pathway FAs are directly integrated into the glycerol backbones within the thylakoid membranes. Another way to combine FAs with PA, the so-called eukaryotic pathway, takes place in the membranes of the endoplasmic reticulum (ER). DAGs synthesized within the ER are subsequently transported to the chloroplast to be converted into GGLs. Recently it was determined that under physiological conditions in *A. thaliana* approximately one half of the plastidic GGLs is formed via the prokaryotic pathway and the other half is synthesized within the eukaryotic pathway. However, the main representative of the thylakoid GPs, namely PG, is predominantly derived from the prokaryotic pathway. Moreover, *A. thaliana*, like about 12% of the Angiosperm species, belongs to the so-called 16:3 plants, i.e., plants, which under physiological growth conditions, contain more than 10% of the total MGDG pool with 16:3 and 18:3 FA residues (Higashi and Saito, 2019). Plant mutants, with decreased levels of 16:3 or 16:3 and 18:3 FAs, appeared to be more sensitive to low temperatures and expressed growth inhibition and leaf chlorosis at 6 but not at 22°C (Hölzl and Dörmann, 2019). Numerous plant species, including wheat and turf grasses, in which the prokaryotic pathway is not used for the synthesis of GGLs, are termed 18:3 plants. In the thylakoids of these plants GGLs are synthesized via the eukaryotic pathway and thus contain 18:3 at the sn-2 position, while GGLs with 16:3 FAs can only be found in trace amounts (Roughan and Slack, 1984; Mongrand et al., 1998; Higashi and Saito, 2019). Additionally, nowadays 468 plant species are known, whose leaf FA profile suggests a loss of the prokaryotic pathway during evolution (Mongrand et al., 1998). Moreover, in plants possessing both active pathways of PA acylation, the temperature was shown to play an important role in the balance between the prokaryotic and eukaryotic pathways. Decreases of the expression of important genes of the prokaryotic pathway during heat stress lead to reduced levels of 16:3 FAs in MGDG and DGDG at the sn-2 position (Higashi et al., 2015; Li et al., 2015). In addition, FAD8, which introduces double bonds at the ω-3 position of the saturated acyl chains of the MGDG molecule, is degraded under high temperatures (Matsuda et al., 2005). Heat-stress also leads to a hydrolization of the 18:3 FAs of MGDG by a chloroplast heat-inducible lipase (HIL1), which in this way initiates and contributes to the MGDG degradation under high temperatures. The concerted action of these mechanisms explains the decreased levels of MGDG and DGDG-bound polyunsaturated FAs (PUFAs) during heat stress (Higashi et al., 2018). Interestingly, in chloroplasts, the decrease of 18:3 FAs is accompanied by an increased concentration 18:2 acyl chains in the GGLs (Higashi and Saito, 2019).

Temperature is not the sole environmental factor resulting in modification of the FA composition of the thylakoid lipids. Thylakoid membrane lipids and the FAs bound to the lipids are involved in the protection against a great number of biotic and abiotic stresses generated by various environmental factors (Wang et al., 2014). Among the thylakoid lipids, MGDG plays a central role in these protection mechanisms. MGDG is highly enriched in 18:3 and 16:3 PUFAs whereas the other membrane lipids DGDG, SQDG, and PG also contain saturated FAs in the form of 16:0. MGDG seems to be involved in the protection against reactive oxygen species (ROS) and it has been suggested that MGDG molecules surrounding photosystem (PS) I and II act as efficient scavengers of ^1^O_2_ as well as hydroxyl radicals which are mainly created within PSII (Schmid-Siegert et al., 2016). MGDG is furthermore engaged in a cyclic mechanism with antioxidative function. In this process lipid peroxidation products such as malondialdehyde (MDA) are formed within the GGL molecules, which, after self-regeneration, can again be fragmented into MDA. This way, the PUFAs bound to the thylakoid membrane GGLs act as a sink for various types of ROS (Mène-Saffrané et al., 2009; Schmid-Siegert et al., 2016). Moreover, 18:3 FAs are postulated to act as direct non-enzymatic scavengers of ROS (Mène-Saffrané et al., 2009).

Besides the formation of MDA, oxidative stresses, including heat and high light stress, lead to a conversion of the lipid-bound PUFAs to lipid peroxides (LOOHs). This lipid peroxidation generates different types of harmful chemical components such as the secondary products of lipid peroxidation and ROS. Lipid peroxidation products subsequently cause protein cleavage, protein oxidation or crosslinking between proteins, lipids and proteins with lipids. The numerous molecules which provide protection against or are formed by the action of ROS and lipid peroxidation products within the thylakoid membrane include (i) large complexes, like the central PSII dimers which dissociate from the surrounding LHCs, (ii) smaller molecules, like the photodamaged D1 protein together with degraded zexanthin expoxidase (ZEP, see section Xanthophyll Cycles of Higher Plants and Algae), and finally (iii) small molecules, like the de-epoxidized xanthophylls of the xanthophyll cycle (see section Xanthophyll Cycles of Higher Plants and Algae). All of these molecules and processes require a free movement in the thylakoid membrane to exert their protective function (Jahns et al., 2009; Kirchhoff, 2014; Yamamoto, 2016). Free movement in the thylakoid membrane depends on the fluidity of the membrane which itself is determined by the structure and composition of the FAs bound to the thylakoid membrane lipids. Highly unsaturated FAs lead to a membrane in a more fluid state whereas a high concentration of saturated FAs increases the rigidity of the thylakoids. Thus, changes of the FA composition of thylakoid membrane lipids in response to various environmental stresses seem to play an important role in the remodeling and functional stabilization of the membranes and the integrated protection mechanisms. With regard to the FA composition it has become clear that photoautotrophs inhabiting different ecosystems show pronounced differences in the FA profiles of both MGDG and the other thylakoid lipids. While in general the FA composition of the thylakoid lipids of the majority of freshwater green algae is comparable to that of 16:3 plants, the algal lipids seem to be enriched in 16C PUFAs while the C18 PUFAs exhibit lower concentrations. On the other hand, marine green algae are rich in C18 PUFAs, whereas red algae show high contents of C20 PUFAs, such as the 20:5 eicosapentaenoic acid (EPA) or the 20:4 arachidonic acid (AA). High levels of both C18 and C20 PUFAs are also typical for brown algae (Goss and Wilhelm, 2009; Kumari et al., 2013).

In diatoms MGDG contains the main long-chain FA of diatoms, i.e., eicosapentaenoic acid (EPA, 20:5, Yongmanitchai and Ward, 1993; Yan et al., 2011; Dodson et al., 2013; Dodson et al., 2014). EPA is preferentially bound to the sn-1 position of the glycerol backbone whereas the sn-2 position is usually occupied by C16 FAs with varying degrees of unsaturation (16:1, 16:2, 16:3, 16:4). MGDG with C20:5 and C16:3 seems to represent the most abundant form of the diatom GGL. Besides the C20/C16 forms of MGDG, MGDG molecules with C16 FAs at both the sn-1 and sn-2 position can be observed. DGDG exhibits a comparable FA composition to MGDG and is also enriched in EPA (Yongmanitchai and Ward, 1993; Yan et al., 2011; Dodson et al., 2013, 2014). Like in the MGDG molecule EPA usually occupies the sn-1 position of DGDG while at the sn-2 position C16 FAs are observed. As it has been demonstrated for MGDG, DGDG forms with C16 FAs at both the sn-1 and sn-2 positions can be found and DGDG with C20:5 and C16:3 seems to represent the most abundant diatom DGDG molecule. Earlier studies have reported that a difference exists in the FA composition of centric and pennate diatoms and that in the pennate diatoms EPA is replaced by C18 FAs in the MGDG and DGDG molecules (Dodson et al., 2013). However, more recent studies have indicated that the differences in the FA composition of MGDG and DGDG of centric and pennate diatoms may be related to differences in their reaction to environmental temperatures and that EPA is present at lower temperatures and may be replaced by C18 FAs at higher temperatures (Dodson et al., 2014). The anionic membrane lipid SQDG of diatoms seems to be enriched in FAs with a shorter chain length and C14 and C16 FAs are usually observed in both the sn-1 and sn-2 positions (Yongmanitchai and Ward, 1993; Yan et al., 2011). The second anionic lipid PG, on the other hand, seems to contain C18:1 FAs as the main molecular species (Yan et al., 2011).

The FA composition of MGDG also influences the three-dimensional structure of the MGDG molecule (see section Three Dimensional Structures of Lipids). Among the thylakoid lipids, MGDG represents the sole non-bilayer lipid and forms the so-called inverted hexagonal phases (H_II_-phases) in aqueous solutions. The ability of MGDG to form H_II_-phases as well as the properties of the H_II_-phase, such as the flexibility, strongly depend both on the proportion of MGDG in a lipid mixture and the FA composition of the MGDG molecule. High concentrations of MGDG-bound PUFAs seem to facilitate the H_II_ formation (Kobayashi et al., 2016).

### Three Dimensional Structures of Lipids

All lipids can be divided into two groups depending on the type of lipid phases they create in aqueous systems. These groups are the bilayer-forming and the non-bilayer-forming lipids. Lipids of the first group aggregate to bilayers, which form lamellar phases in one of the two main states: (i) “fluid” lamellar liquid crystalline (L_α_) phases, also referred to as liquid-disordered (L_d_, L_l/d_) phases, or (ii) “solid” lamellar gel (L_β_) phases, which are also designated as ordered solid (S_o_) phases. The second group of lipids includes non-bilayer-forming lipids which can aggregate to normal or inverted (reversed) phases which are denoted with subscripts I and II, respectively. Normal phases, which are commonly observed in neutral lipid/water systems, are micellas (L_1_), normal discontinuous cubic phases (I_I_), normal hexagonal phases (H_I_) and the normal bicontinuous cubic phases (Q_I_). The reversed phases include inverted micellas (L_2_), reversed bicontinuous cubic phases (Q_II_), the reversed discontinuous cubic phases (Q_III_), and the inverted hexagonal phases (H_II_) (Huang and Gui, 2018). Types of aggregates, as well as types of phases created by the lipids, are determined both by geometry of the lipid molecule and several physicochemical parameters of its surroundings. The chemical geometry of the lipid molecule is described by the critical packing parameter (CPP) which denotes the ratio of the maximum volume, which can be occupied by the FA residues (V), and the product of the length of these residues (l) and the cross-sectional area of the hydrophilic lipid headgroups (a) (Table 1; Yamashita, 2018). The relatively small polar headgroup of the MGDG molecule, accompanied by the large area occupied by the expanded PUFA tails, results in a cone-like geometry of the molecule. This structure enables MGDG to spontaneously form H_II_-phases in both aqueous systems and model or natural lipid membranes (Shipley et al., 1973). H_II_-phases consist of a great number of tightly packed cylindrical micelles with a diameter between 1–2 nm and contain 30–60 weight percent of water. DGDG, SQDG and PG possess a cylindrical shape due to the large headgroup areas and the lower content of longer-chain PUFAs and thus belong to the bilayer-forming lipids (Jouhet, 2013). Besides the influence of the inherent chemical structures of the lipid molecules, the formation of lipid phases is strongly affected by the neighboring molecules like proteins, pigments, other lipids or even ions. Divalent cations, for example, facilitate the formation of H_II_-phases. With respect to the lipid phases formed by MGDG it has been demonstrated that the proteins involved in the phototransformation of protochlorophylls support the formation of a cubic phase (Selstam, 1998). For the function of the thylakoid membrane it is of high importance that the LHCII strongly interacts with MGDG and forces the MGDG molecules into a membrane bilayer structure (Simidjiev et al., 2000). In thylakoid membranes, MGDG seems to play a key role in providing the membrane fluidity which is essential for the efficient diffusion of the xanthophll cycle pigments (see section Xanthophyll Cycles of Higher Plants and Algae), LHC proteins or proteins involved in the turnover and repair of PSII and ZEP. Thus, MGDG and the H_II_-phases created by MGDG are not only important for chlorophyll biosynthesis during chloroplast development (Jarvis et al., 2000), but also play significant role in the proper functioning of the photosynthetic machinery in differentiated chloroplasts (Zhou et al., 2009; Schaller et al., 2011). On a molecular level MGDG molecules have been shown to be integral parts of the PSI and PSII core complexes (van Eerden et al., 2017) where they promote the PSII dimerization or form a cavity for the docking of plastoquinone Q_B_ (Guskov et al., 2009; Kansy et al., 2014). However, the importance of the three-dimensional structures formed by MGDG for the function of the MGDG molecules located within the PSI and PSII core complexes remains to be clarified.

**TABLE 1:** Dependence of the lipid self-assembly structures on the value of the critical packing parameter (CPP).

**Critical packing parameter value (CPP)** $\begin{array}{c} CPP\;=\;V/a*l\\ a\\ v\}l \end{array}$	**Type of structure**
≤1/3	Normal micelle (L_1_), (I_I_)
[1/3−1/2]	Normal hexagonal phase (H_I_)
[1/2−1]	Normal bicontinuous cubic phase (Q_I_)
≈ 1	Lamellar phases (L_α_, L_d_, L_β_)
≥1	Reversed bicontinuous cubic phase (Q_II_)
>1	Reversed micelle (L_2_) Reversed discontinuous cubic (Q_III_) Reversed hexagonal phase (H_II_)

*V, volume, which can be occupied by the FA residues; l, length of the FA residues; a, the cross-sectional area of the hydrophilic lipid headgroups. For further detail see Yamashita (2018).*

Recently, the results of an *in silico* study using the PSII x-ray structure of the thermophilic cyanobacterium *Thermosynechococcus vulcanus* showed that lipid domains surrounding the PSII core complex may be also enriched in SQDG. Like it was discussed above for MGDG, it is not clear if and how the three-dimensional phases formed by SQDG influence the PSII structure and function (van Eerden et al., 2017).

With respect to the H_II_-phases formed by MGDG it was demonstrated that physical factors, like a high temperature or dehydration, can also induce the formation of non-lamellar phases. Low pH-values, as they are typical for the thylakoid lumen during high light illumination have also been shown to promote the formation of H_II_-phases by MGDG (Garab et al., 2017, see also section Localization of Non-bilayer Lipid Phases in the Thylakoid Membrane).

## Xanthophyll Cycles of Higher Plants and Algae

### Types of Xanthophyll Cycles

**FIGURE 2:**
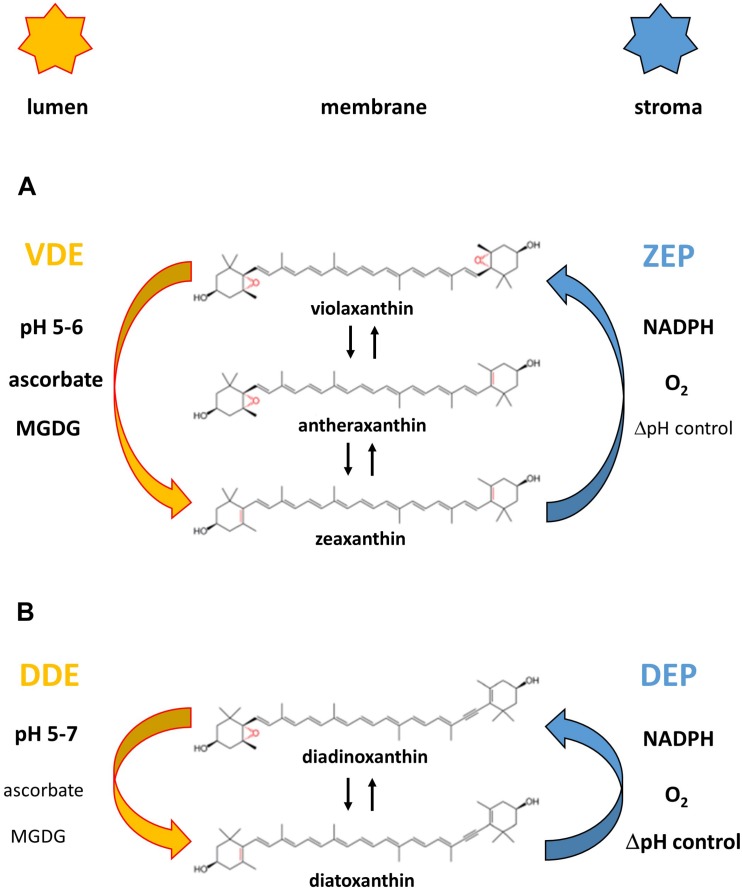
Reaction sequences and enzymes of the violaxanthin **(A)** and diadinoxanthin **(B)** cycle. The violaxanthin cycle is present in higher plants and green and brown algae, the diadinoxanthin cycle is found in diatoms, haptophytes and dinophytes. Figure 2 also shows the cofactor requirements of the enzymes catalyzing the de-epoxidation reaction (violaxanthin de-epoxidase, VDE and diadinoxanthin de-epoxidase, DDE) and the epoxidation reaction (zeaxanthin epoxidase, ZEP and diatoxanthin epoxidase, DEP), respectively. Representation of the cofactors in bold or normal type indicates whether high or low concentrations of the respective cofactors are needed for high enzyme activity. The establishment of a proton gradient inhibits diatoxanthin epoxidation (high ΔpH control) and is thus presented in bold type whereas zeaxanthin epoxidation is unaffected by the presence of the transmembrane ΔpH (ΔpH control depicted in normal type). The pH value of the thylakoid lumen which leads to VDE and DDE activation (possibly by VDE or DDE dimerization) and membrane binding is also indicated for the two xanthophyll cycles.

The main xanthophyll cycles that are known today are the violaxanthin (Vx) cycle of higher plants, green and brown algae and the diadinoxanthin (Ddx) cycle of diatoms, haptophytes and dinophytes (Figure 2, for a review see Goss and Jakob, 2010). Algae containing the Ddx cycle also contain the pigments of the Vx cycle because Vx is a precursor in the biosynthesis pathway of the Ddx cycle pigments (Lohr and Wilhelm, 1998, 2001). The presence of Vx cycle pigments in Ddx cycle containing algae is especially visible during longer periods of high light exposure when a pronounced *de novo* synthesis of Ddx cycle pigments is taking place. In addition to these two xanthophyll cycles, the existence of a lutein epoxide (Lx) cycle has been shown which is, however, restricted to some families of higher plants (Esteban and Garcia-Plazaola, 2014). Some members of the Prasinophyceae, which represent a class of unicellular green algae, or some species within the genus *Gracilaria* belonging to the Rodophyta are characterized by a modified Vx cycle (Goss et al., 1998; Bertrand and Schoefs, 1999; Cardol et al., 2008).

### Reaction Sequences and Xanthophyll Cycle Enzymes

The xanthophyll cycles consist of forward reactions which take place during illumination of plants or algae with high light illumination and back reactions which revert the forward reaction during periods of low light exposure or darkness. In the Vx cycle the forward reaction consists of a two-step de-epoxidation of the di-epoxy xanthophyll Vx to the mono-epoxide Ax and finally to the epoxy-free Zx (Yamamoto et al., 1962; Hager, 1967a). The back reaction re-introduces the two epoxy groups into the Zx molecule and regenerates Vx via the intermediate reaction product Ax (Hager, 1967a). The Vx cycle of the Prasinophyceae and of some species of the genus *Gracilaria* is incomplete and during high light illumination an accumulation of the intermediate de-epoxidation product Ax is observed in Prasinophycean cells (Goss et al., 1998; Cardol et al., 2008). In the red algae *Graciliaria gracilis* or *G. multipartite* an absence of Vx was detected and Ax seems to be the only substrate which can be converted to Zx (Bertrand and Schoefs, 1999). Despite the ongoing uncertainties about the presence of a xanthophyll cycle in Rodophyta, a recent study has presented clear evidence that at least a Zx epoxidase, which is able to convert Zx to Ax, is present in the red algae (Dautermann and Lohr, 2017).

Thus, these two modifications of the Vx cycle, like the Ddx and the Lx cycles, consist of only one de-epoxidation and one epoxidation step. In the Vx cycle of the Prasinophyceae the xanthophyll di-epoxide Vx is converted to the mono-epoxide Ax whereas in all other one-step xanthophyll cycles mono-epoxides such as Ax, Ddx and Lx are converted to the epoxy-free xanthophylls such as Zx, diatoxanthin (Dtx), and lutein (L), respectively (Hager and Stransky, 1970; Stransky and Hager, 1970; Rabinowitch et al., 1975; Bungard et al., 1999; Bertrand and Schoefs, 1999). In the back reaction one epoxy-group is re-introduced into the epoxy-free xanthophylls Dtx or L and Ddx or Lx are regenerated. The forward reaction of the Vx cycle is catalyzed by the enzyme Vx de-epoxidase (VDE), the back reaction by the enzyme Zx epoxidase (ZEP). Both the VDE and the ZEP belong to a family of diverse proteins, the so-called lipocalins (Hieber et al., 2000; Grzyb et al., 2006). Lipocalins usually bind hydrophobic molecules and act as carrier proteins for their substrates. VDE is localized in the thylakoid lumen and has a pH-optimum of around pH 5.2 (Hager, 1969; Pfündel et al., 1994). During high light illumination the light-driven proton gradient leads to a decrease of the pH-value of the thylakoid lumen, thereby activating the VDE. Activation of VDE is probably driven by a dimerization of the water-soluble monomeric VDE, followed by the binding of the active, dimeric VDE to the lumen side of the thylakoid membrane (Hager and Holocher, 1994; Arnoux et al., 2009; Saga et al., 2010). With regard to the dimerization it has been proposed that the C-terminus of the VDE plays a role in the interaction of the VDE monomers (Hallin et al., 2016) and that four specific amino acid residues are important for the pH-dependent activation (Fufezan et al., 2012). In addition, it has been suggested that the conserved cysteine residues and the disulfide bridges formed by the cysteines are sensitive to redox changes of the thylakoid membrane induced by the photosynthetic electron transport (Hallin et al., 2015; Simionato et al., 2015). Changes of the thylakoid redox potential may play a role in the regulation and activation of the VDE via dithiol/disulfide exchange reactions. Reduced ascorbate has been identified as the co-substrate of the de-epoxidation reaction and is important for the reduction of the epoxy group and the subsequent abstraction as H_2_O (Hager, 1969). Interestingly, a recent study has reported that the atypical VDE of the green algae *Chlamydomonas reinhardtii* (Li et al., 2016) does not require the presence of ascorbate (Vidal-Meireles et al., 2020). In contrast to the VDE the ZEP seems to be permanently associated with the stromal side of the thylakoid membrane as a peripheral membrane protein (Schaller et al., 2012b). ZEP uses O_2_ and NADPH + H^+^ as co-substrates to re-introduce the epoxy group into the Zx and Ax molecule, respectively (Hager, 1967a). The VDE of higher plants and green algae is characterized by a higher substrate affinity for Ax compared with Vx which results in faster kinetics of the second de-epoxidation step from Ax to Zx compared with the first de-epoxidation step from Vx to Ax (Frommolt et al., 2001; Goss, 2003). However, the VDE of the Prasinophyceae differs from the VDE of higher plants and other green algae and exhibits a higher substrate affinity for Vx compared with Ax (Goss, 2003). This results in a very slow second de-epoxidation step from Ax to Zx and together with the simultaneous epoxidation reaction results in the accumulation of Ax during high light illumination in these green algae (Frommolt et al., 2001). Interestingly, the decreased substrate affinity of the VDE of the Prasinophyceae is not restricted to the mono-epoxide Ax but the enzyme is characterized by a generally low substrate affinity for xanthophyll mono-epoxides whereas xanthophyll di-epoxides like Vx are converted with high efficiency. For the Lx cycle which occurs in some families of higher plants it has been suggested that the VDE and ZEP also carry out the additional de-epoxidation of Lx to L and from L back to Lx (Esteban et al., 2009). The enzymes of the Ddx cycle in diatoms show some differences to the respective enzymes in higher plants and green algae. VDEs in diatoms and some other groups of algae, like dinophytes, haptophytes or phaeophytes, are also denoted as diadinoxanthin de-epoxidases (DDEs). Before 2007 the presence of different VDE genes and thus the presence of different VDEs/DDEs in organisms containing the Ddx cycle was not known and the measurements dealing with the properties of the DDE were ascribed to one single enzyme. It was shown that the DDE is characterized by a pH-optimum which is shifted toward higher pH-values (Jakob et al., 2001). In addition, it has been reported that DDE activity and thus the de-epoxidation of Ddx to Dtx can take place at neutral pH-values. In addition, DDE is able to operate efficiently with lower concentrations of the co-substrate ascorbate compared with the VDE of higher plants (Grouneva et al., 2006).

It might be possible that a relationship exists between the higher SQDG and lower MGDG concentrations of the diatom thylakoid membrane (see section Lipid Classes) and the broad pH-optimum of the diatom DDE. Taking into account that the thylakoid membranes of the *A. thaliana mgd1-1* mutant, which are strongly reduced in their MGDG content, are impaired in their ability to build-up a strong proton gradient during illumination (Aronsson et al., 2008, see also section Lipid Classes), a comparable situation might occur in the diatom thylakoids with their low MGDG concentration. The possible inability of the diatom thylakoid membranes to generate a very strong ΔpH would then require the onset of DDE activity at a weaker pH-gradient across the membrane and thus at higher luminal pH-values. Such a behavior, i.e., DDE activity at almost neutral pH-values, has been observed in *in vitro* experiments where the pH-activity and pH-optimum of the DDE were determined (Jakob et al., 2001).

Today, it is clear that the diatom genome codes for more than one de-epoxidase. Two DDE-encoding genes were shown to be present in the centric diatom *Thalassiosira pseudonana* (Montsant et al., 2007). One of these genes (labeled as *TpVDE*) is similar to the genes encoding the typical plant VDEs while the other (designated as violaxanthin de-epoxidase-like; *TpVDL*) is more distantly related. Later, it was demonstrated that the genome of the pennate diatom *Phaeodactylum tricornutum* contains one gene similar to the genes of “typical VDEs” (termed *PtVDE*) and two VDE-like genes, designated as *PtVDL1* and *PtVDL2* (Coesel et al., 2008). Important differences can also be observed for the epoxidation reaction of the Ddx cycle. Here it has been demonstrated that the Dtx epoxidase (DEP) shows significantly higher Dtx epoxidation rates than the ZEP of higher plants and green algae (Mewes and Richter, 2002; Goss et al., 2006). The kinetics of the conversion of Dtx to Ddx are almost comparable to the very fast de-epoxidation of Ddx to Dtx by the DDE. To avoid a futile cycle and to enable a fast accumulation of Dtx during illumination with high light intensities the DEP underlies a strict light-dependent control (Mewes and Richter, 2002; Goss et al., 2006). DEP activity is completely suppressed during high light illumination by the build-up of the light-driven proton gradient. During low light illumination or during periods of darkness when no or only a weak trans-membrane proton gradient is present, DEP is fully activated and Dtx is rapidly converted back to Ddx.

### Localization of Xanthophyll Cycle Pigments in the Thylakoid Membrane

With respect to the localization of the xanthophyll cycle pigments in the thylakoid membrane two main pools can be differentiated. The first pool consists of xanthophyll cycle pigments which are bound to the light-harvesting proteins. In higher plants and green algae the majority of the protein-bound Vx cycle pigments is associated with the light-harvesting complex of photosystem II (LHCII), which represents the main thylakoid membrane protein (Ruban et al., 1999), although proteins of the light-harvesting complex of photosystem I (LHCI) also bind Vx, as well as L and β-carotene (Croce et al., 2002, 2007). Interestingly, recent data have shown that the binding of the xanthophyll cycle pigments can have an impact on the supramolecular structure of LHCII (Zhou et al., 2020). LHCII contains a special binding site for Vx which has been termed V1. V1 is located at the periphery of each LHCII apoprotein and contains a loosely bound Vx molecule (Morosinotto et al., 2003). In the minor PSII antenna proteins CP29, CP26, and CP24, which contain higher concentrations of Vx cycle pigments compared to the LHCII, Vx seems to occupy the L2 binding site (Morosinotto et al., 2003). This binding site is usually responsible for the association of one of the two lutein molecules in the LHCII. In contrast to the V1 site the L2 site is not located at the periphery but represents an internal pigment binding site. Experiments with recombinant LHCII and minor PSII antenna proteins have shown that only Vx, which is associated with the peripheral V1 binding site, can be efficiently converted to Ax and Zx (Jahns et al., 2001; Wehner et al., 2006). Vx bound to the internal L2 binding site is not easily accessible by the VDE and no or only a very limited de-epoxidation can be observed. The loose association of Vx with the LHCII apoprotein at the peripheral binding site is thought to enable the efficient detachment of Vx from the protein during high light illumination followed by the diffusion into the lipid phase of the thylakoid membrane where the actual enzymatic conversion to Ax and Zx by the enzyme VDE is taking place (Latowski et al., 2002; Goss et al., 2007). Recently, it has been shown that the binding of neoxanthin (Nx) to the LHCII affects the binding affinity of Vx (Wang et al., 2017). In the presence of Nx Vx is only weakly bound to the LHCII, easily dissociates into the lipid phase of the membrane, thereby enhancing the first step of the de-epoxidation reaction. In contrast to the general assumption that Zx rebinds to the Vx binding sites at the LHCII and the minor PSII antenna proteins after the de-epoxidation of Vx, Xu et al. (2015) provided evidence that Zx does not necessarily exchange for Vx in the internal binding sites. It may be located in the periphery of the complexes and exert its quenching capacity in a position between the LHCs.

In diatoms the protein bound Ddx cycle pigments are located in the different fucoxanthin chlorophyll protein (FCP) complexes. The recent elucidation of the molecular structure of an FCP complex composed of Lhcf3 and Lhcf4 by x-ray crystallography (Wang et al., 2019) showed that, like Vx in the LHCII, Ddx is located at the periphery of the apoprotein and thus most likely also loosely bound. Like the easy detachment of Vx from the LHCII followed by the rebinding of Zx, the peripheral binding of Ddx is thought to facilitate the exchange with Dtx during the operation of the Ddx cycle. Interestingly, the binding site of the Ddx molecule seems to be located at the opposite side of the apoprotein compared with the Vx binding site in the LHCII. Besides the Lhcf proteins which build-up the peripheral antenna system of diatoms, but may also be more closely associated with the PSII core complex, the Lhcr proteins which form the PSI-specific antenna of diatoms, bind Ddx cycle pigments. According to Lepetit et al. (2008) the concentration of Ddx and Dtx seems to be even slightly higher in the PSI antenna compared to the peripheral FCP complex. Like higher plants and green algae diatoms show an increase of the xanthophyll cycle pigment pool upon cultivation with higher light intensities (Lavaud et al., 2003; Schumann et al., 2007; Gundermann and Büchel, 2008; Lepetit et al., 2010; Gundermann et al., 2019). The increase of the Ddx cycle pigment pool size is significantly more pronounced compared with the increase of the Vx cycle pigment concentrations. With respect to the additional Ddx and Dtx synthesized during exposure to high light intensities it has been proposed that a part of these additional Ddx cycle pigments is bound by the photoprotective Lhcx proteins which also show a stronger expression under high light conditions (Lepetit et al., 2013). In the centric diatoms which are characterized by a more complicated antenna system than the pennate diatoms, it could be shown that both the FCPa and FCPb complexes bind Ddx cycle pigments (Gundermann and Büchel, 2008). In the FCPa complex increased Ddx binding during high light cultivation was accompanied by an increased content of the Fcp6 and Fcp7 proteins. Interestingly, Dtx-induced decreases of the FCP fluorescence emission could only be observed in the FCPa.

It is noteworthy that the differences in the main thylakoid membrane proteins, i.e., the light-harvesting complexes (LHCs), go together with differences in the lipid and FA composition of the thylakoids (see sections Lipid Classes and Fatty Acids). In higher plants and green algae, which contain the LHCII and LHCI, the thylakoid lipids are rich in C16 and C18 FAs. Diatoms and brown algae, which are characterized by the presence of Fx, contain MGDG and DGDG molecules with a high concentration of C18 and C20 FAs. It may be possible that the differences in the FA composition of MGDG and DGDG between the green lineage and diatoms/brown algae are related to differences in the structures of LHCs and FCPs. Furthermore, these differences may represent an optimal harmonization of the thylakoid membranes to allow the best possible LHC/FCP structure and function, i.e., light-harvesting or non-photochemical quenching of Chl a fluorescence (NPQ, see section Function of Xanthophyll Cycles). With respect to the different xanthophyll cycles in higher plants/green algae and diatoms the differences in the lipid and FA composition may even influence the interplay between the LHCs/FCPs and the xanthophyll cycle enzymes. In this regard, it is interesting that the Vx cycle enzymes of brown algae, which contain FCP complexes and are enriched in C18 and C20 FAs, show some of the typical features of the Ddx cycle enzymes of diatoms, like e.g., a fast epoxidation reaction (Garcia-Mendoza and Colombo-Pallotta, 2007).

The second main pool of xanthophyll cycle pigments consists of Vx or Ddx cycle pigments which are not protein bound but localized as free pigments in the lipid phase of the thylakoid membrane. First evidence for the existence of free Zx molecules was obtained from studies on the fluidity of thylakoid membranes which showed that the conversion of Vx to Zx leads to a rigidification of the membrane (Gruszecki and Strzalka, 1991, 2005; Tardy and Havaux, 1997, see also section Function of Xanthophyll Cycles). Isolation of LHCII with different preparation methods led to the purification of LHCII with different concentrations of native lipids and Vx cycle pigments (Schaller et al., 2010). Further analysis of the LHCII preparations demonstrated that the concentration of LHCII-associated Vx was correlated with the amount of MGDG which was isolated with the complexes. Decreases of the MGDG content led to a decrease of the Vx concentration, indicating that a part of the Vx cycle pigment pool was protein-bound whereas another part was localized within an MGDG-shield surrounding the LHCII. Comparable results were obtained for the Prasinophyceae *Mantoniella squamata* where LHC could be isolated which contained high concentrations of MGDG and Vx (Schaller et al., 2012a). In diatoms which are characterized by a strong increase of the Ddx cycle pigment pool during high light exposure a comparable separation between a protein bound and lipid dissolved Ddx cycle pigments could be observed. The first indication for a pool of Ddx cycle pigments, which are not bound to FCP complexes, was obtained from measurements of NPQ of high light cultivated diatoms (Schumann et al., 2007). These measurements indicated that additional Dtx synthesized during high light treatment is not able to enhance NPQ and thus cannot be bound to the respective binding sites of the FCP complexes. Additional experiments with isolated FCP complexes from low and high light cultivated diatom cells demonstrated that the additional Ddx cycle pigments show the same absorption spectrum as purified Ddx which is dissolved in MGDG (Lepetit et al., 2010). The enrichment of MGDG in the isolated FCP complexes led to the conclusion that, like in higher plants, the diatom antenna complexes are surrounded by an MGDG shield which incorporates a part of the Ddx cycle pigments. Also like in higher plants the free Ddx cycle pigments have been shown to play a role in the modulation of the thylakoid membrane fluidity (Bojko et al., 2019, see section Function of Xanthophyll Cycles).

### Function of Xanthophyll Cycles

The xanthophyll cycles of plants and algae are important protection mechanisms against damage to the photosynthetic apparatus induced by high light intensities. The function of the xanthophyll cycles as photoprotective mechanisms is trifold: (i) they play an important role in the dissipation of excessive excitation energy as heat in the process of NPQ (Goss and Lepetit, 2015; Ruban, 2016), (ii) they are able to directly scavenge ROS within the thylakoid membrane (Havaux, 1998; Triantaphylidès and Havaux, 2009) and (iii) they serve as stabilizers of the lipid phase of the thylakoid membrane (Gruszecki and Strzalka, 2005; Bojko et al., 2019).

With respect to the process of NPQ the de-epoxidized xanthophylls Zx and Dtx have been shown to induce a structural change of the light-harvesting complexes of higher plants (Ruban et al., 1992, 1997; Holzwarth et al., 2009) and diatoms (Gundermann and Büchel, 2008; Miloslavina et al., 2009; Schaller-Laudel et al., 2015). This structural change leads to the transformation of excessive excitation energy into heat followed by the harmless dissipation of thermal energy. In higher plants protonation of the LHCII and the presence of the PsbS protein (Li et al., 2000) are further essential factors which regulate NPQ and thus the structural change of the PSII antenna. In this respect, Welc et al. (2016) have shown that Vx cycle pigments which are not bound to the LHCII can nonetheless modulate the structure of the LHCII. Vx seems to promote the formation of LHCII supramolecular structures whereas free Zx induced an LHCII structure suitable for the dissipation of excessive excitation energy. Recently, an interaction between Zx and the PsbS protein has been described which leads to a preferential association of PsbS with the minor PSII antenna proteins (Sacharz et al., 2017). PsbS itself seems to form clusters and may act as initiator for LHCII aggregation. LHCII aggregation can also be induced *in vitro* by the addition of Mg^2+^ ions (Schaller et al., 2014) which in the chloroplast act as counter-ions to the light-induced proton influx from the stroma to the thylakoid lumen during the build-up of the transmembrane proton gradient. In green algae comparable LHCII aggregation has been observed during the induction of NPQ, the role of the PsbS protein in the establishment of NPQ has, however, been replaced by the LHCSR proteins (Peers et al., 2009; Bonente et al., 2011; Gerotto and Morosinotto, 2013). Like PsbS the LHCSR3 protein has been proposed to sense the decrease of the pH in the thylakoid lumen during illumination (Ballottari et al., 2016). Nawrocki et al. (2019) have shown that LHCSR proteins are responsible for NPQ in *Chlamydomonas reinhardtii* but that PsbS proteins also play a role in photoprotection. LHCII aggregation is characterized by a shift of the chlorophyll a fluorescence emission to longer wavelengths (Holzwarth et al., 2009) which, interestingly, can also be observed upon the aggregation of FCP complexes in diatoms (Miloslavina et al., 2009; Lavaud and Lepetit, 2013). In diatoms the Lhcx proteins have been demonstrated to adopt the role of the PsbS protein (Bailleul et al., 2010; Zhu and Green, 2010; Taddei et al., 2016, 2018). Recently, it was demonstrated that NPQ depends on the concerted action of the Ddx cycle and the Lhcx proteins and that Lhcx proteins provide photoprotection via the thermal dissipation of excitation energy (Buck et al., 2019). For both higher plants (Holzwarth et al., 2009; Jahns and Holzwarth, 2012) and diatoms (Goss and Lepetit, 2015; Taddei et al., 2018) models for the localization and function of NPQ have been established. These models predict the formation of two quenching sites where the transformation of excitation energy into heat is taking place. Quenching site Q1 is composed of detached LHCII and FCP complexes, respectively, which after their dissociation from the PSII core complex undergo a structural change and form aggregates. NPQ at quenching site Q1 seems to be independent of the de-epoxidation of Vx to Zx. For the formation of quenching site Q2, however, the presence of Zx is important. Quenching site Q2 is located in the vicinity of the PSII core complex and most likely involves the minor PSII antenna proteins in higher plants and green algae and special FCP proteins which are more closely associated with the PSII core in diatoms. Newer data indicates that NPQ in trimeric LHCIIs does depend on Zx but not on lutein whereas NPQ in the monomeric LHC proteins requires Zx and L and involves the formation of a radical pair (Dall’Osto et al., 2017). Recently, it has been demonstrated that PsbS-dependent NPQ occurs mainly in the LHCII whereas another quenching site operates within the PSII core complex (Nicol et al., 2019). For plants containing the Lx cycle and green algae exhibiting the shortened Vx/Ax cycle it was demonstrated that L or Ax can play a similar role in NPQ induction and enhancement as it is normally observed for Zx (Goss et al., 1998; Garcia-Plazaola et al., 2003; Leonelli et al., 2017).

While the xanthophyll cycle-dependent induction of NPQ is linked to Zx or Dtx bound to the antenna proteins of higher plants and algae, the anti-oxidative function of Zx or Dtx is related to those molecules which are localized as free pigment in the lipid phase of the membrane. For both Zx and Dtx it has been shown that these xanthophylls are able to detoxify ROS which are generated by alternative electron pathways or via the triplet excited state of Chl a under supersaturating light conditions (Havaux and Niyogi, 1999; Havaux et al., 2007; Lepetit et al., 2010). Deactivation of ROS by Zx or Dtx minimizes the damaging effects of ROS on membrane lipids, the embedded photosynthetic membrane proteins and the photosynthetic pigments. The antioxidant function of Zx may be located at the interface between LHCII and the membrane lipids because it was shown that Zx associated with oligomeric LHCII is active in the detoxification of ROS (Johnson et al., 2007).

Recently, a further function of de-epoxidized xanthophyll cycle pigments has been proposed. From former studies it was known that the conversion of Vx to Zx increases the membrane rigidity of thylakoids (Gruszecki and Strzalka, 1991, 2005). The membrane stabilizing effect of Zx was attributed to the fact that Zx spans the complete thylakoid membrane and that the polar head groups of the xanthophyll molecule are anchored within the hydrophilic part of the membrane where the lipid head groups are located (Gruszecki and Strzalka, 2005, see also Grudzinski et al., 2017). Furthermore, it was proposed that the xanthophyll molecules play a comparable role in the modulation of the physical membrane properties as cholesterol in animal or human membranes. The recent analysis of the effect of Dtx on membrane properties provided additional information on the action of the de-epoxidized xanthophylls (Bojko et al., 2019). Based on EPR measurements with the 5-SASL and 16-SASL spin probes it was shown that during the conversion of Ddx to Dtx a dynamic effect takes place whereas the high Dtx concentrations after de-epoxidation exert a stable effect on the properties of the diatom thylakoid membrane. The combined action of both effects results in a temporary increase of the rigidity of both peripheral and central parts of the membrane bilayer whereas the stable effect leads to a more permanent increase of the rigidity of the hydrophobic core of the membrane. Both effects are supposed to play a role in the short-term adaptation of diatom thylakoid membranes to changing temperatures.

## Lipid Dependence of Xanthophyll Cycling in Higher Plants and Algae

### Lipids as Solvents for Xanthophyll Cycle Pigments

First evidence for the role of lipids in the process of Vx de-epoxidation was obtained by Yamamoto et al. (1974) and Yamamoto and Higashi (1978) who isolated VDE which contained MGDG as single lipid component and VDE without attached MGDG. In *in vitro* enzyme assays with the isolated VDEs it became clear that MGDG is needed for the de-epoxidation of Vx to Ax and Zx and that a ratio of MGDG to Vx of about 30 is ideal for the efficient conversion. The authors concluded that one of the functions of MGDG is the solubilization of the hydrophobic pigment, thereby presenting the substrate in a form that meets the requirements of the active site of the VDE. More recent experiments with single lipids have shown that membrane lipids which form inverted hexagonal structures, i.e., MGDG and phosphatidylethanolamine (PE), have a high capacity to solubilize the xanthophyll cycle pigments Vx and Ddx (Latowski et al., 2004; Goss et al., 2005). Membrane lipids like DGDG, SQDG or PC, which form bilayers in aqueous solutions, are also able to solubilize Vx or Ddx but significantly higher concentrations are needed to achieve complete solubilization. Solubilization converts aggregates of the hydrophobic pigments Vx or Ddx into lipid dissolved single molecules which can then be de-epoxidized by the enzyme VDE. The higher capacity of the non-bilayer lipids MGDG and PE to solubilize the xanthophyll cycle pigments can also be seen in artificial membranes which are composed of a non-bilayer and bilayer lipid (Goss et al., 2007). Liposomes constructed with equal amounts of PC/PE, PC/MGDG or DGDG/MGDG show a strong enrichment of Vx or Ddx in non-bilayer lipids. The preferential localization of the xanthophyll cycle pigments in the liposome non-bilayer phase is in line with the presence of Vx or Ddx in the MGDG shield surrounding the higher plant or diatom antenna complexes (Lepetit et al., 2010; Schaller et al., 2010). Taken together this indicates that in the native membrane the xanthophyll cycle pigments are enriched in non-bilayer lipid phases. With respect to the comparison of the two main xanthophyll cycle pigments Vx and Ddx the experiments demonstrated that higher concentrations of inverted hexagonal phase forming lipids are needed for the complete solubilization of Vx compared with Ddx (Goss et al., 2005). The increased solubility of Ddx in MGDG is in line with the decreased concentration of MGDG and the increased amount of Ddx cycle pigments in the diatom thylakoid membrane compared with the thylakoids of higher plants and green algae (Lavaud et al., 2003; Gundermann and Büchel, 2008; Lepetit et al., 2010, 2012). Complete solubilization of Ddx and Dtx under these conditions can only be achieved if a low lipid per pigment ratio is sufficient for solubilization. While in the studies detailed above no or only a very slow conversion of Vx or Ddx to Zx or Dtx was observed upon the complete solubilization of the substrates in bilayer lipids, comparable experiments performed by Yamamoto (2006) yielded slightly different results. These results indicated that MGDG or DGDG support Vx de-epoxidation in different ways. While the presence of MGDG leads to a fast and complete conversion of Vx to Zx, the de-epoxidation is slow but nevertheless complete when DGDG is added to the enzyme assay. Based on the results it was concluded that the solubilization of aggregated Vx by DGDG proceeds during the time course of the de-epoxidation reaction thereby circumventing the negative effects of the decreased solubilization capacity of the bilayer forming lipid.

### Role of Non-bilayer Lipid Phases for Xanthophyll Cycling

The first results on the role of inverted hexagonal phases for xanthophyll de-epoxidation were reported by Latowski et al. (2000, 2002, 2004). The authors used unilamellar liposomes composed of PC, which were supplemented with different concentrations of the non-bilayer lipid MGDG, to study the conversion of Vx to Ax and Zx. In the liposome systems an increase in the de-epoxidation efficiency was observed with increasing ratios of MGDG to PC. In addition, through the use of ^31^P-NMR spectroscopy, the presence of inverted hexagonal phases formed by MGDG was detected. Based on the results it was concluded that Vx diffuses into the inverted hexagonal phase where the actual conversion to Ax and Zx by the enzyme VDE is taking place. The importance of inverted hexagonal phases for Vx de-epoxidation was demonstrated in a subsequent study where different non-bilayer and bilayer lipids where tested for their ability to enhance the de-epoxidation reaction (Latowski et al., 2004). The non-bilayer lipids MGDG and PE induced a fast and efficient conversion of Vx whereas in the presence of the bilayer lipids DGDG and PC no or only a very slow Vx de-epoxidation could be observed. The use of either GGLs or GPs demonstrated that not the nature of the lipid but the three-dimensional structures formed by the lipids are responsible for the stimulation of Vx de-epoxidation. Later it could be shown that the bilayer lipids DGDG or PC are able to completely solubilize either Vx or Ddx (Goss et al., 2005). However, no or only a very slow conversion of Vx to Ax and Zx or Ddx to Dtx was observed in the single bilayer lipid systems. In liposome systems composed of only bilayer lipids, i.e., DGDG or PC, a complete solubilization of Vx or Ddx could be achieved as well, but Vx or Ddx de-epoxidation could only be detected when the liposomes where supplemented with a certain concentration of the inverted hexagonal phase forming lipids MGDG or PE (Goss et al., 2007). Later it was demonstrated that the main lipid of diatom thylakoid membranes, the negatively charged SQDG has a pronounced inhibitory effect on Ddx de-epoxidation (Goss et al., 2009). A comparable inhibition of the DDE could be demonstrated for the anionic GP PG. The results from the solubilization and de-epoxidation experiments in single lipid systems and liposomes composed of different lipids implied that the solubilization of the lipids represents an important factor for the de-epoxidation of Vx or Ddx but that, despite efficient solubilization, the three-dimensional structures of the lipids are mandatory for an efficient conversion of the xanthophyll cycle pigments. Based on these results it was suggested that the inverted hexagonal structure and not the bilayer structure enables the access of the enzymes VDE or DDE to their respective solubilized substrates. The penetration of the VDE or DDE into the hydrophobic interior of the lipid phase has to be deep enough to allow the interaction of the catalytic site of the enzymes with the substrates Vx or Ddx. Penetration of the β-barrel structure, which forms the catalytic site of the lipocalins to which both VDE and DDE belong (Hieber et al., 2000), may be facilitated by a decreased surface tension of the inverted hexagonal phase compared with the bilayer phase (van den Brink-van der Laan et al., 2004). The decreased surface tension is most probably caused by the conical shape of the non-bilayer lipids like MGDG molecules which are characterized by a small headgroup and long unsaturated FAs which cover a significantly larger area than the mono-galactose headgroup (Garab et al., 2000, see section Three Dimensional Structures of Lipids). Bilayer lipids like DGDG, on the other hand, have a cylindrical shape because the larger headgroups and FA chains cover a comparable area. Lipid phases composed of bilayer lipids are thus rather tightly sealed, show a high surface tension and do not enable the penetration of VDE or DDE into the hydrophobic membrane interior. Yamamoto (2006) proposed a different concept of Vx de-epoxidation in the thylakoid membrane. Based on the observation that Vx could be completely converted to Zx in the presence of the bilayer lipid DGDG, albeit with a significantly lower Vx de-epoxidation rate compared to MGDG, it was proposed that Vx de-epoxidation is not restricted to MGDG reverse micelles and that the VDE is able to operate throughout the lipid phase of the single bilayer thylakoid membrane.

### Localization of Non-bilayer Lipid Phases in the Thylakoid Membrane

The existence of non-bilayer lipid phases in thylakoid membranes has been studied by freeze-fracture electron microscopy. It was demonstrated that exposure of higher plant thylakoid membranes to increasing temperatures in the range from 35 to 45°C leads to a destacking of grana membranes (Gounaris et al., 1984). Further increases of the temperature up to 45–55°C induces a pronounced lipid phase separation and the formation of large areas of inverted hexagonal lipid phases. Lipid phase separation and irreversible induction of inverted hexagonal phases can also be triggered by exposure of thylakoid membranes to pH-values lower than pH 4.5 or treatment with phospholipase A_2_ (Thomas et al., 1985). In addition, incubation of isolated thylakoids in reaction buffers complemented with high concentrations of compatible solutes, such as sucrose, trehalose, sorbitol or betaine, induces the phase separation of non-bilayer forming lipids, followed by the establishment of large areas of inverted hexagonal lipid phases (Williams et al., 1992). Later, first measurements of isolated wheat thylakoid membranes with ^31^P-NMR spectroscopy supported the formation of inverted hexagonal lipid phases when the thylakoids were exposed to high temperatures between 55 and 60°C (Haranczyk et al., 1995). Krumova et al. (2008) used PG as an intrinsic bulk label lipid for ^31^P-NMR studies to analyze the lipid phases of the thylakoid membrane. The data showed that besides the lamellar phase a non-bilayer isotropic phase exists which becomes predominant at higher temperatures. In addition, it was demonstrated that the phospholipid was not restricted to the lamellar phase of the membrane. Using molecular dynamics simulations for the characterization of the thylakoid membranes of higher plants and cyanobacteria differences between these membranes could be detected (van Eerden et al., 2015). The simulations revealed that the thylakoid membrane is in a state close to the formation of the inverted hexagonal phase. Furthermore, the molecular dynamic simulations showed that the higher plant thylakoid membrane more readily undergoes the transition to the inverted hexagonal phase compared with the cyanobacterial membrane which is most likely caused by the higher degree of unsaturation of the FA moieties of the plant lipids. It is interesting to note that the simulations of van Eerden et al. (2015) did not present evidence for an enrichment of MGDG molecules in the inverted hexagonal phase. Instead, a well-mixed system of lipids could be observed in both the lamellar and the inverted hexagonal phase. Recent studies employing ^31^P-NMR measurements and time-resolved fluorescence spectroscopy of the fluorescent, lipophilic dye MC_540_ revealed the presence of not only one but three inverted hexagonal phases which coexist with one bilayer phase in the thylakoid membrane of higher plants (Garab et al., 2017). According to the data the three inverted hexagonal phases are located at the luminal and stromal sides of the thykakoid membrane, accompanied by a lipid phases in the junction region of the grana and stroma membranes. Garab et al. (2017) showed that the non-bilayer lipid phases were sensitive to the osmolarity and the ionic strength of the medium which was later confirmed by the results of Kotakis et al. (2018) who used high concentrations of the compatible solute sucrose to induce the formation of the inverted hexagonal phases. Besides the presence of osmolytes low pH-values led to a pronounced increase of the non-bilayer thylakoid lipid phases which could additionally be modulated by the degree of unsaturation of the lipid FAs.

### Localization of Non-bilayer Phases Involved in Xanthophyll Cycling

It has been proposed that the non-bilayer lipid phases involved in xanthophyll cycling are localized in the vicinity of the LHCII in higher plants and green algae or the FCP complexes in diatoms. This proposal is based on the purification of LHCII and FCP complexes which, under mild solubilization conditions, leads to the isolation of LHCII and FCP complexes with a shield of MGDG molecules incorporating the Vx or Ddx cycle pigments (see section Localization of Xanthophyll Cycle Pigments in the Thylakoid Membrane and references therein). In addition, a fast and efficient rebinding of the de-epoxidized xanthophyll cycle pigments Zx or Dtx to the light-harvesting complexes has to take place in order to establish rapid xanthophyll-dependent photoprotection via NPQ (section Function of Xanthophyll Cycles and references). With respect to the binding of the pigment molecules to the LHC apoproteins it is interesting to note that the de-epoxidized Ddx cycle pigment Dtx shows a decreased solubility in MGDG compared with the epoxidized Ddx which possibly accelerates the rebinding of Dtx to the FCP complex (Goss et al., 2007). Since in higher plants and green algae the majority of the Vx cycle pigments are bound to the LHCII (section Localization of Xanthophyll Cycle Pigments in the Thylakoid Membrane and references) it is reasonable to believe that most of the non-bilayer lipid phases are located in the vicinity of LHCII and thus in the grana regions of the thylakoid membrane. On the other hand, Vx de-epoxidation is not restricted to the LHCII but also occurs in the LHCI (Lee and Thornber, 1995; Arvidsson et al., 1997; Zhu et al., 1997) which would require the presence of inverted hexagonal phases in the stroma thylakoids in the vicinity of LHCI. However, based on the observation that Vx de-epoxidation in the LHCI is less efficient than that in LHCII (Wehner et al., 2004) the non-bilayer lipid phases in the stroma thylakoid membranes may be less well-defined as the non-bilayer lipid phases in the grana membranes. The idea of a tight association of the non-bilayer lipid phase and the light-harvesting complexes is supported by the analysis of the molecular structure of the LHCII at 3.2 Å resolution which revealed the presence of a putative binding site for the enzyme VDE (Liu et al., 2004). It is thus possible that the local non-bilayer lipid phase enables the direct interaction between the VDE and the LHCII. A close association between the LHCII and the VDE is furthermore corroborated by the recent direct isolation of a functional Vx cycle membrane domain from thylakoid membranes of higher plants (Goss et al., 2017). With the use of the very mild detergent α-dodecylmaltoside and a preparation at pH5, which ensured the binding of VDE to the luminal side of the thylakoid membrane, the authors were able to isolate a membrane domain consisting of LHCII, VDE and MGDG. The domain was in functional state as evidenced by the de-epoxidation of Vx to Ax and Zx by the domain-associated VDE. However, the studies of Arvidsson et al. (1997) and Macko et al. (2002) are in favor of a greater distance between the LHCII and the lipid phase of the membrane where the actual Vx de-epoxidation is taking place. In both studies the activity of isolated, exogenous VDE was analyzed. VDE was added to the stromal side of thylakoid membranes where the activity of the endogenous VDE at the luminal site of the membrane was inhibited by DTT (Arvidsson et al., 1997) or to the stromal side of thylakoids isolated from *Arabidopsis thaliana* mutants that did not contain VDE (Macko et al., 2002). Both studies show that the de-epoxidation efficiency of VDE located at the stromal side of the membrane is comparable to the activity of native VDE at the luminal side of the thylakoid membrane. Since in these experiments the VDE located at the stromal membrane site had no accession to the grana stacks it was concluded that the lipid phase where de-epoxidation is taking place is located at a certain distance to the central part of the grana membranes and located at the surface of the grana stacks. This notion was supported by the lower VDE activity at reduced temperatures which the authors interpreted in such a way that Vx cycle pigments, which have detached from the LHCII, have to diffuse a certain distance in the membrane until they reach the lipid phase for de-epoxidation.

With respect to the possible localization of the non-bilayer lipid phase in diatom thylakoids it has to be taken into account that the diatom thylakoid membranes show a different architecture compared with the thylakoids of higher plants. The diatom thylakoids are not separated into grana and stroma thylakoids but are characterized by regular stacks of three membranes (Gibbs, 1962, 1970). Based on the observations that MGDG forms a lipid shield around the FCP complexes which incorporates the lipid-dissolved Ddx cycle pigments (Lepetit et al., 2010) and that the negatively charged lipid SQDG, which is present in high concentration in the diatom membranes, inhibits the de-epoxidation of Ddx (Goss et al., 2009) a model for the lipid and protein arrangement in the diatom thylakoid membranes was established (Lepetit et al., 2012). This model predicts that PSII with its tightly associated FCP complexes and its peripheral antenna complexes is located in the inner membranes of the stacks of three membranes. In addition, the inner core membranes are enriched in the PSII-associated MGDG. The outer membranes of the regular stacks contain the PSI complexes with their PSI-specific FCP complexes and the ATP synthase. According to the model, the lipid composition of the outer membranes is dominated by the anionic SQDG. Recently, the model of Lepetit et al. (2012) was supported by new data which show that the thylakoid membranes of the pennate diatom *P. tricornutum* contain large areas which are exclusively occupied by PSI supercomplexes, consisting of PSI core complexes with their specific antenna complexes (Bina et al., 2016). Flori et al. (2017) also obtained evidence for a compartmentalization of the two photosystems. Like the model of Lepetit et al. (2012) they propose that PSII is located in the core membranes whereas PSI is enriched in the peripheral membranes which are exposed to the chloroplast stroma. Since the FCP complexes associated with PSII are surrounded by an MGDG phase, MGDG is most probably also enriched in the inner membranes. The enrichment of MGDG in the core membranes may lead to the formation of non-bilayer structures which then represent attraction sites for the diatom DDE and support the efficient conversion of Ddx to Dtx. The confinement of SQDG to the outer, peripheral membranes is most likely needed in order to prevent the interaction with the DDE in the non-bilayer phases in the core membranes which would result in a pronounced inhibition of Ddx de-epoxidation.

### Formation of Non-bilayer Lipid Phases by Structural Changes of Light-Harvesting Proteins

**FIGURE 3:**
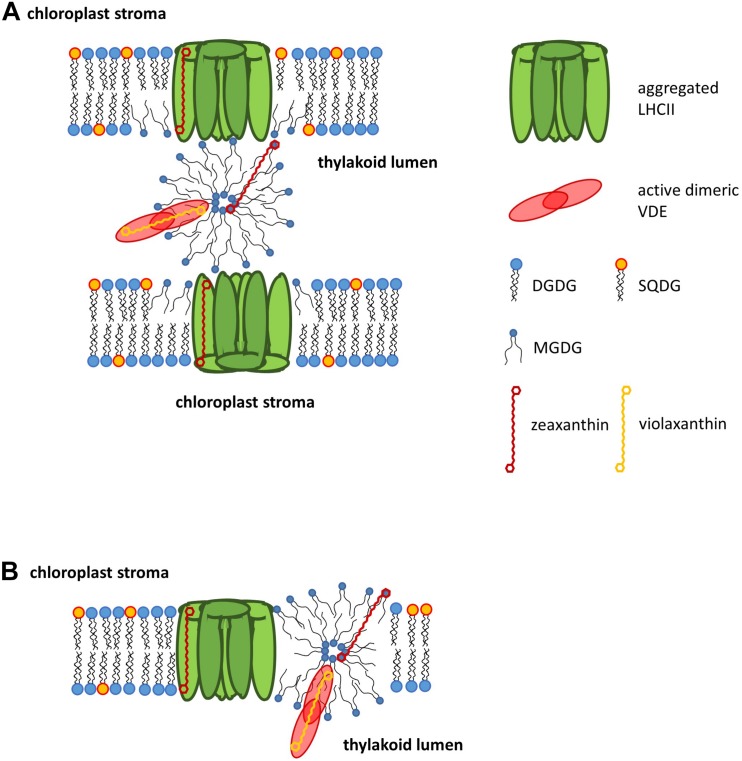
Model for the violaxanthin cycle domain in thylakoid membranes of higher plants. High light illumination leads to a disconnection of the main light-harvesting complex of photosystem II, LHCII from the photosystem II core complex followed by protein aggregation. MGDG molecules, which during low light illumination surround the LHCII, dissociate, segregate and form a non-bilayer lipid phase. This non-bilayer lipid phase may be located outside of the membrane bilayer, i.e., within the thylakoid lumen **(A)**, or within the plane of the thylakoid membrane **(B)**. During high light illumination violaxanthin disconnects from its binding site at the LHCII apoproteins and diffuses into the non-bilayer lipid phase. The non-bilayer lipid phase represents an attraction site for the enzyme violaxanthin de-epoxidase (VDE), which, after its pH-dependent activation and dimerization, binds to the non-bilayer lipid phase. The non-bilayer lipid phase is characterized by a reduced surface tension and thus allows the penetration of the enzyme’s hydrophobic catalytic site into the hydrophobic core of the non-bilayer lipid phase where it gains access to the hydrophobic pigment violaxanthin. After the conversion of violaxanthin to zeaxanthin, zeaxanthin rebinds to the LHCII and participates in photoprotection via NPQ. Since the violaxanthin cycle domain, consisting of LHCII, MGDG, VDE and xanthophyll cycle pigments, is only established during high light illumination of plants, the domain can be described as a transient membrane domain.

The high concentration of MGDG in thylakoid membranes of higher plants and green algae poses the risk of the formation of inverted hexagonal phases and the segregation of MGDG out of the membrane bilayer in an aqueous environment. In the native thylakoids the formation of extensive non-bilayer lipid phases is restricted by the high protein content of the membranes and extensive lipid phase separation can only be observed when thylakoids are exposed to various abiotic stresses. For the thylakoids of higher plants it has been demonstrated that the interaction of the main membrane protein LHCII and MGDG plays a crucial role in establishing and maintaining the lipid bilayer structure (Simidjiev et al., 1998, 2000). *In vitro* studies with isolated LHCII and MGDG showed that in the presence of MGDG large, ordered lamellar structures of protein and lipid are formed. In addition, the presence of LHCII disturbs the inverted hexagonal phase of MGDG and forces the MGDG molecules into a bilayer structure. Based on these results it was proposed that in thylakoid membranes the spatial limitation caused by the high concentration of membrane proteins inhibits the formation of non-bilayer lipid phases. Additional studies demonstrated that MGDG stabilizes the oligomeric states of LHCII (Schaller et al., 2011), FCP complexes and the LHC of the Prasinophyceae *M. squamata* (Schaller-Laudel et al., 2017). Furthermore, MGDG increases the mechanical stability of the LHCII (Seiwert et al., 2017), thereby preventing the unfolding of trimeric LHCII. Stabilization of LHCII by MGDG was attributed to a steric matching of the conically shaped MGDG and the hourglass shape of trimeric LHCII (Seiwert et al., 2017). Several studies have shown that LHCII in its lipid environment is structurally flexible and can undergo molecular rearrangements (see section Function of Xanthophyll Cycles and references therein). During high light illumination of plants or algae, which triggers the process of NPQ, aggregation of LHCII is taking place. With respect to the formation of non-bilayer lipid phases in the thylakoid membrane the aggregation of LHCII or FCP complexes is thought to play an important role. Protein aggregation, in general, leads to a segregation of lipid molecules which are otherwise associated with the non-aggregated protein complexes. It is thought that in the case of the LHCII and FCP complexes aggregation leads to the liberation of a significant part of the MGDG molecules associated with the light-harvesting proteins followed by a separation from the lipid bilayer phase of the thylakoid membrane and finally the formation of inverted hexagonal phases. Although it is still a matter of debate if the non-bilayer phases are formed within the lipid bilayer (Jahns et al., 2009) or are associated with the outside of the membrane (Garab et al., 2016 see Figure 3), recent results have implicated that non-bilayer lipid phases are located at the luminal and stromal sides of the thylakoid membrane (Garab et al., 2017).

### The Xanthophyll Cycle Membrane Domain in the Light of Recent Membrane Models

More than 40 years ago the fluid mosaic model of biomembranes was introduced by Singer and Nicholson (1972). The first main feature of this model is the amphiphilic nature of the membrane lipids which means that the lipids contain a hydrophilic head group and a hydrophobic area covered by the FA moieties (see section Three Dimensional Structures of Lipids). The amphiphilic character of the lipids leads to the formation of a lipid bilayer in aqueous surroundings. A second important feature concerns the proteins which can be associated with the hydrophilic head groups of the lipids as peripheral proteins or with the hydrophobic core of the membrane as trans-membrane proteins. A third important aspect lies in the fluidity of the membrane which means that both lipids and proteins are in constant motion. Protein and lipid motion can occur in lateral direction, i.e., in the plane of the membrane. In addition, rotation of proteins and lipids along their long axis is possible. Since the first presentation of the fluid mosaic model a wealth of information about the structure of biomembranes has been gathered (for recent reviews see Bagotelli and Mouritsen, 2013; Goni, 2014; Nicholson, 2014). Although these data do not question the general assumptions of the fluid mosaic model they have refined the model in several important ways. The first important aspect concerns the high concentration of membrane proteins. While in the original model only few proteins span the membrane bilayer it is known today that the membrane is densely packed with membrane and peripheral proteins (for a review see Engelmann, 2005). Especially in membranes like the thylakoid membrane or the inner mitochondrial membrane the protein density is so high that bilayer phases composed solely of lipids are hardly observed (for a recent review on the thylakoid architecture see Kirchhoff, 2018). A second new finding, which is also critical for the understanding of the xanthophyll cycle function, is the observation that proteins exist which only transiently bind to biomembranes (reviewed by Goni, 2002). This class of proteins can exist in water-soluble form in an aqueous environment but can also bind to the membrane after certain requirements have been met. The transient proteins supplement the proteins which are permanently associated with the membrane and the water-soluble proteins which never come into contact with the lipid bilayer. A further important extension of the original fluid mosaic model, which is also of fundamental importance for the operation of the xanthophyll cycle, is the finding that non-bilayer phases can co-exist with the lipid bilayer (for the presence of inverted hexagonal phases in thylakoid membranes see section Localization of Non-bilayer Lipid Phases in the Thylakoid Membrane and references therein). Another feature which separates the new membrane models from the original fluid mosaic model concerns the observation that biomembranes are characterized by a pronounced lateral heterogeneity. It has become clear that the membrane bilayer consists of heterogeneous domains (for reviews on domains and lipid rafts in plant membranes see Mongrand et al., 2010; Simon-Plas et al., 2011). These membrane domains contain a specific lipid and protein composition and perform a special function. Other important aspects of the new generation of membrane models describe the membrane not as a flat surface but as a curved structure. In this regard the observation of a hydrophobic mismatch is interesting (for a review see Killian, 1998). Hydrophobic mismatch describes the situation when the hydrophobic thickness of the transmembrane region of a membrane protein does not match the hydrophobic thickness of the membrane in which it is localized. Here it has been shown that both membrane proteins, by re-arrangements of the protein structure, and membrane lipids, by stretching or tilting of the FA chains, can adapt to this situation. According to the results outlined in the preceding chapters (see references therein) the part of the thylakoid membrane where the Vx cycle, and most likely the Ddx cycle, too, takes place can be described as a special membrane domain (Figure 3). The Vx cycle domain contains a specific protein composition, namely the LHCII, a membrane protein which is permanently associated with the membrane, and the VDE, a water-soluble protein which, after its pH-dependent activation, transiently binds to the membrane. The Vx cycle domain is characterized by a specific lipid composition because it is enriched in the non-bilayer lipid MGDG which likely forms an inverted hexagonal structure within the domain. The enrichment of MGDG is driven by the structural re-arrangement, i.e., the aggregation, of the LHCII which leads to a local enrichment of free MGDG molecules. The Vx cycle domain carries out a special function, i.e., the conversion of the di-epoxy xanthophyll Vx to Ax and Zx. The Vx cycle domain can be characterized as a transient domain which is only established during high light illumination when Vx de-epoxidation is needed for photoprotection via NPQ. A transition from high light illumination back to low light conditions or darkness reverses the domain formation by a detachment of the VDE from the membrane and a disaggregation of LHCII. Disaggregation of the LHCII re-enables the tight interaction of the membrane protein with MGDG, thereby re-enforcing the membrane bilayer structure.

## Outlook

Although recent measurements have begun to shed light on the existence and localization of inverted hexagonal phases in the thylakoid membrane of higher plants (Garab et al., 2017), and the isolation of a putative, functional Vx cycle membrane domain has been reported (Goss et al., 2017), there is still a wealth of open questions with respect to the lipid dependence of xanthophyll cycling. Future measurements have to show where the inverted hexagonal phase responsible for Vx de-epoxidation is located, i.e., whether it is located within the plane of the thylakoid membrane or whether it is attached to the luminal side of the membrane. With respect to the localization of the non-bilayer lipid phase it has to be clarified if the lipid phase is located in the direct vicinity of the LHCII or at a greater distance, possibly in the grana margins. A greater distance between the xanthophyll cycle pigment binding proteins and the lipid phase, where the actual enzymatic reaction is taking place, would require significantly longer diffusion times of Vx and Zx. Future measurements have to provide a way to determine the size of the inverted hexagonal phases to answer the question if fewer but larger non-bilayer lipid phases exist or if the xanthophyll cycle pigments are converted in a larger number of smaller sized inverted hexagonal phases. In this respect it has to be demonstrated if the VDE, which seems to be present in only very low concentrations in the thylakoid lumen (Arvidsson et al., 1996), stays attached to a larger non-bilayer phase or if it detaches and rebinds to a higher number of smaller phases. With regard to the conversion of Vx to Ax and Zx in the LHCI (Croce et al., 2002, 2007) it has to be elucidated if non-bilayer phases or even xanthophyll cycle domains exist in the vicinity of PSI and thus the stroma membranes. According to our knowledge it is also unclear if the back reaction of the xanthophyll cycle, which is carried out by the peripheral membrane protein ZEP (Schaller et al., 2012b), requires the presence of special membrane lipid structures, like e.g., the inverted hexagonal phase, and where this hypothetical phase or domain is located. Finally, scientists working with algae may concentrate on the question if similar xanthophyll cycle domains exist in the different algal classes and, taking into account the different thylakoid structures (Lepetit et al., 2012; Flori et al., 2017), where these domains are localized.

## Author Contributions

RG and DL wrote and corrected the text and constructed the figures.

## Conflict of Interest

The authors declare that the research was conducted in the absence of any commercial or financial relationships that could be construed as a potential conflict of interest.

## References

[B1] Andersson M. X. Larsson K. E. Tjellström H. Liljenberg C. Sandelius A. S. (2005). Phosphate limited oat. The plasma membrane and the tonoplast as major targets for phospholipid-to-glycolipid replacement and stimulation of phospholipases in the plasma membrane. *J. Biol. Chem.* 280 27578–27586. 10.1074/jbc.M503273200 15927962

[B2] Andersson M. X. Stridh M. H. Larsson K. E. (2003). Phosphate deficient oat replaces a major portion of the plasma membrane phospholipids with the galactolipid digalactosyldiacylglycerol. *FEBS Lett.* 537 128–132. 10.1016/S0014-5793(03)00109-1 12606044

[B3] Arnoux P. Morosinotto T. Saga G. Bassi R. Pignol D. (2009). A structural basis for the pH-dependent xanthophyll cycle in *Arabidopsis thaliana*. *Plant Cell* 21 2036–2044. 10.1105/tpc.109.068007 19638474 PMC2729612

[B4] Aronsson H. Schüttler M. A. Kelly A. A. Sundqvist C. Dörmann P. Karim S. (2008). Monogalactosyldiacylglycerol deficiency in *Arabidopsis thaliana* affects pigment composition in the prolamellar body and impairs thylakoid membrane energization and photoprotection in leaves. *Plant Physiol.* 148 580–592. 10.1104/pp.108.123372 18641085 PMC2528128

[B5] Arvidsson P.-O. Bratt C. E. Carlsson M. Akerlund H.-E. (1996). Purification and identification of the violaxanthin deepoxidase as a 43 kDa protein. *Photosynth. Res.* 49 119–129. 10.1007/bf00117662 24271609

[B6] Arvidsson P.-O. Carlsson M. Stefansson H. Albertsson P. A. Akerlund H.-E. (1997). Violaxanthin accessibility and temperature dependency for de-epoxidation in spinach thylakoid membranes. *Photosynth. Res.* 52 39–48.

[B7] Bagotelli L. A. Mouritsen O. G. (2013). Is the fluid mosaic (and the accompanying raft hypothesis) a suitable model to describe fundamental features of biological membranes? What may be missing? *Front. Plant Sci.* 4:457 10.3389/fpls.2013.00457 PMC3826152 24312108

[B8] Bailleul B. Rogato A. de Martino A. Coesel S. Cardol P. Bowler C. (2010). An atypical member of the light-harvesting complex stress-related protein family modulates diatom responses to light. *Proc. Natl. Acad. Sci. U.S.A.* 107 18214–18219. 10.1073/pnas.1007703107 20921421 PMC2964204

[B9] Ballottari M. Truong T. B. De Re E. Erickson E. Stella G. R. Fleming G. R. (2016). Identification of pH-sensing sites in the light-harvesting complex stress-related 3 protein essential for triggering non-photochemical quenching in *Chlamydomonas reinhardtii*. *J. Biol. Chem.* 291 7334–7346. 10.1074/jbc.m115.704601 26817847 PMC4817166

[B10] Bassi R. Pineau B. Dainese P. Marquardt J. (1993). Carotenoid-binding proteins of photosystem II. *Eur. J. Biochem.* 212 297–303. 10.1111/j.1432-1033.1993.tb17662.x 8444169

[B11] Ben Hamed K. Ben Youssef N. Ranieri A. Zarrouk M. Abdelly C. (2005). Changes in content and fatty acid profiles of total lipids and sulfolipids in the halophyte *Crithmum maritimum* under salt stress. *J. Plant Physiol.* 162 599–602. 10.1016/j.jplph.2004.11.010 15940877

[B12] Bertrand M. Schoefs B. (1999). “Photosynthetic pigment metabolism in plants during stress,” in *Handbook of Plant And Crop Stress*, 2nd Edn, ed. Pessarakli M. (New York, NY: Marcel Dekker), 527–541.

[B13] Bina D. Herbstova M. Gardian Z. Vacha F. Litvin R. (2016). Novel structural aspect of the diatom thylakoid membrane: lateral segregation of photosystem I under red-enhanced illumination. *Sci. Rep.* 6:25583. 10.1038/srep25583 PMC4857733 27149693

[B14] Bojko M. Olchawa-Pajor M. Goss R. Schaller-Laudel S. Strzalka K. Latowski D. (2019). Diadinoxanthin de-epoxidation as important factor in the short-term stabilization of diatom photosynthetic membranes exposed to different temperatures. *Plant Cell Environ.* 42 1270–1286. 10.1111/pce.13469 30362127

[B15] Bonente G. Ballotari M. Truong T. B. Morosinotto T. Ahn T. K. Fleming G. R. (2011). Analysis of LhcSR3, a protein essential for feedback de-excitation in the green alga *Chlamydomonas reinhardtii*. *PLoS Biol.* 9:e1000577 10.1371/journal.pbio.1000577 PMC3022525 21267060

[B16] Boudiere L. Michaud M. Petroutsos D. Rébeillé F. Falconet D. Bastien O. (2014). Glycerolipids in photosynthesis: composition, synthesis and trafficking. *Biochim. Biophys. Acta* 1837 470–480. 10.1016/j.bbabio.2013.09.007 24051056

[B17] Buck J. M. Sherman J. Bartulos C. R. Serif M. Halder M. Henkel J. (2019). Lhcx proteins provide photoprotection via thermal dissipation of absorbed light in the diatom *Phaeodactylum tricornutum*. *Nat. Commun.* 10:4167. 10.1038/s41467-019-12043-6 PMC6744471 31519883

[B18] Bungard R. A. Ruban A. V. Hibberd J. M. Press M. C. Horton P. Scholes J. D. (1999). Unusual carotenoid composition and a new type of xanthophyll cycle in plants. *Proc. Natl. Acad. Sci. U.S.A.* 96 1135–1139. 10.1073/pnas.96.3.1135 9927706 PMC15363

[B19] Cardol P. Bailleul B. Rappaport F. Derelle E. Béal D. Breyton C. (2008). An original adaptation of photosynthesis in the marine green alga *Ostreococcus*. *Proc. Natl. Acad. Sci. U.S.A.* 105 7881–7886. 10.1073/pnas.0802762105 18511560 PMC2409423

[B20] Chen J. Burke J. J. Xin Z. Xu C. Velten J. (2006). Characterization of the Arabidopsis thermosensitive mutant atts02 reveals an important role for galactolipids in thermotolerance. *Plant Cell Environm.* 29 1437–1448. 10.1111/j.1365-3040.2006.01527.x 17080965

[B21] Coesel S. Obornik M. Varela J. Falciatore A. Bowler C. (2008). Evolutionary origins and functions of the carotenoid biosynthetic pathway in marine diatoms. *PLoS One* 3:e2896 10.1371/journal.pone.0002896 PMC2483416 18682837

[B22] Croce R. Morosinotto T. Castelletti S. Breton J. Bassi R. (2002). The Lhca antenna complexes of higher plants photosystem I. *Biochim. Biophys. Acta* 1556 29–40. 10.1016/s0005-2728(02)00304-3 12351216

[B23] Croce R. Mozzo M. Morosinotto T. Romeo A. Hienerwadel R. Bassi R. (2007). Singlet and triplet state transitions of carotenoids in the antenna complexes of higher-plant Photosystem I. *Biochemistry* 46 3846–3855. 10.1021/bi602531k 17326666

[B24] Dall’Osto L. Cazzaniga S. Bressan M. Palecek D. Zidek K. Niyogi K. K. (2017). Two mechanisms for dissipation of excess light in monomeric and trimeric light-harvesting complexes. *Nat. Plants* 3:17033. 10.1038/nplants.2017.33 28394312

[B25] Dautermann O. Lohr M. (2017). A functional zeaxanthin epoxidase from red algae shedding light on the evolution of light-harvesting carotenoids and the xanthophyll cycle in photosynthetic eukaryotes. *Plant J.* 92 879–892. 28949044 10.1111/tpj.13725

[B26] Demmig B. Winter K. Krüger A. Czygan F.-C. (1987). Photoinhibition and zeaxanthin formation in intact leaves. A possible role for the xanthophyll cycle in the dissipation of excess light. *Plant Physiol.* 84 218–224. 10.1104/pp.84.2.218 16665420 PMC1056560

[B27] Demmig B. Winter K. Krüger A. Czygan F.-C. (1988). Zeaxanthin and the heat dissipation of excess light energy in *Nerium oleander* exposed to a combination of high light and water stress. *Plant Physiol.* 87 17–24. 10.1104/pp.87.1.17 16666096 PMC1054692

[B28] Demmig-Adams B. Adams W. W. III (2006). Photoprotection in an ecological context: the remarkable complexity of thermal energy dissipation. *New Phytol.* 172 11–21. 10.1111/j.1469-8137.2006.01835.x 16945085

[B29] Demmig-Adams B. Koh S.-C. Cohu C. M. Muller O. Stewart J. J. Adams W. W. III (2014). “Non-photochemical fluorescence quenching in contrasting plant species and environments,” in *Non-Photochemical Quenching And Energy Dissipation In Plants, Algae And Cyanobacteria*, eds Demmig-Adams B. Garab G. Adams W. W. Govindjee M. (Dordrecht: Springer), 531–552. 10.1007/978-94-017-9032-1_24

[B30] Dodson V. J. Dahmen J. L. Mouget J.-L. Leblond J. D. (2013). Mono- and digalactosyldiacylglycerol composition of the marennine-producing diatom, *Haslea ostrearia*: comparison to a selection of pennate and centric diatoms. *Phycol. Res.* 61 199–207. 10.1111/pre.12015

[B31] Dodson V. J. Mouget J.-L. Dahmen J. L. Leblond J. D. (2014). The long and short of it: temperature-dependent modifications of fatty acid chain length and unsaturation in the galactolipid profiles of the diatoms *Haslea ostrearia* and *Phaeodactylum tricornutum*. *Hydrobiologia* 727 95–107. 10.1007/s10750-013-1790-4

[B32] Engelmann D. M. (2005). Membranes are more mosaic than fluid. *Nature* 438, 578–580. 10.1038/nature04394 16319876

[B33] Esteban R. Becerril J. M. Garcia-Plazaola J. I. (2009). Lutein epoxide cycle-more than a just a forest tale. *Plant Signal. Behav.* 4 342–344. 10.4161/psb.4.4.8197 19794858 PMC2664502

[B34] Esteban R. Garcia-Plazaola J. I. (2014). “Involvement of a second xanthophyll cycle in non-photochemical quenching of chlorophyll fluorescence: the lutein epoxide story,” in *Non-Photochemical Quenching And Energy Dissipation In Plants, Algae And Cyanobacteria*, eds Demmig-Adams B. Garab G. Adams W. W. (Dordrecht: Springer), 277–295. 10.1007/978-94-017-9032-1_12

[B35] Flori S. Jouneau P. Bailleul B. Gallet B. Estrozi L. F. Moriscot C. (2017). Plastid thylakoid architecture optimizes photosynthesis in diatoms. *Nat. Commun.* 18:15885. 10.1038/ncomms15885 PMC5481826 28631733

[B36] Frentzen M. Heinz E. McKeon T. A. Stumpf P. K. (1983). Specificities and selectivities of glycerol-3- phosphate acyltransferase and monoacylglycerol-3-phosphate acyltransferase from pea and spinach chloroplasts. *Eur. J. Biochem.* 129 629–636. 10.1111/j.1432-1033.1983.tb07096.x 6825679

[B37] Frommolt R. Goss R. Wilhelm C. (2001). The de-epoxidase and epoxidase reactions of *Mantoniella squamata* (Prasinophyceae) exhibit different substrate-specific reaction kinetics compared to spinach. *Planta* 213 446–456. 10.1007/s004250100589 11506368

[B38] Fufezan C. Simionato D. Morosinotto T. (2012). Identification of key residues for pH dependent activation of violaxanthin de-epoxidase from *Arabidopsis thaliana*. *PLoS One* 7:e35669 10.1371/journal.pone.0035669 PMC3338714 22558195

[B39] Garab G. Lohner K. Laggner P. Farkas T. (2000). Self-regulation of the lipid content of membranes by non-bilayer lipids: a hypothesis. *Trends Plant Sci.* 5 489–494. 10.1016/s1360-1385(00)01767-2 11077258

[B40] Garab G. Ughy B. de Waard P. Akhtar P. Javornik U. Kotakis C. (2017). Lipid polymorphism in chloroplast thylakoid membranes - as revealed by ^31^P-NMR and time resolved merocyanine fluorescence spectroscopy. *Sci. Rep.* 7:13343. 10.1038/s41598-017-13574-y PMC5645462 29042649

[B41] Garab G. Ughy B. Goss R. (2016). “Role of MGDG and non-bilayer lipid phases in the structure and dynamics of chloroplast thylakoid membranes,” in *Lipids in Plant and Algae Development*, eds Nakamura Y. Li-Beisson Y. (Dordrecht: Springer), 127–157. 10.1007/978-3-319-25979-6_6 27023234

[B42] Garcia-Mendoza E. Colombo-Pallotta M. F. (2007). The giant kelp *Macrocystis pyrifera* presents a different nonphotochemical quenching control than higher plants. *New Phytol.* 173 526–536. 10.1111/j.1469-8137.2006.01951.x 17244047

[B43] Garcia-Plazaola J. I. Hernandez A. Olano J. M. Becerril J. M. (2003). The operation of the lutein epoxide cycle correlates with energy dissipation. *Funct. Plant Biol.* 30 319–324. 10.1071/FP02224 32689014

[B44] Gerotto C. Morosinotto T. (2013). Evolution of photoprotection mechanisms upon land colonization: evidence of PSBS-dependent NPQ in late Streptophyte algae. *Physiol. Plant.* 149 583–598. 10.1111/ppl.12070 23663155

[B45] Gibbs S. P. (1962). The ultrastructure of the chloroplasts of algae. *J. Ultrastruct. Res.* 7 418–435. 10.1016/s0022-5320(62)90038-2 13947684

[B46] Gibbs S. P. (1970). The comparative ultrastructure of the algal chloroplast. *Ann. N. Y. Acad. Sci.* 175 454–473. 10.1111/j.1749-6632.1970.tb45167.x

[B47] Goni F. M. (2002). Non-permanent proteins in membranes: when proteins come as visitors. *Mol. Membr. Biol.* 19 237–245. 10.1080/0968768021000035078 12512770

[B48] Goni F. M. (2014). The basic structure and dynamics of cell membranes: an update of the singer-nicholson model. *Biochim. Biophys. Acta* 1838 1467–1476. 10.1016/j.bbamem.2014.01.006 24440423

[B49] Goss R. (2003). Substrate specificity of the violaxanthin de-epoxidase of the primitive green alga *Mantoniella squamata* (Prasinophyceae). *Planta* 217 801–812. 10.1007/s00425-003-1044-1 12748855

[B50] Goss R. Böhme K. Wilhelm C. (1998). The xanthophyll cycle of *Mantoniella squamata* converts violaxanthin into antheraxanthin but not to zeaxanthin: consequences for the mechanism of enhanced non-photochemical energy dissipation. *Planta* 205 613–621. 10.1007/s004250050364

[B51] Goss R. Greifenhagen A. Bergner J. Volke D. Hoffmann R. Wilhelm C. (2017). Direct isolation of a functional violaxanthin cycle domain from thylakoid membranes of higher plants. *Planta* 245 793–806. 10.1007/s00425-016-2645-9 28025675

[B52] Goss R. Jakob T. (2010). Regulation and function of xanthophyll cycle-dependent photoprotection in algae. *Photosynth. Res.* 106 103–122. 10.1007/s11120-010-9536-x 20224940

[B53] Goss R. Latowski D. Grzyb J. Vieler A. Lohr M. Wilhelm C. (2007). Lipid dependence of diadinoxanthin solubilization and de-epoxidation in artificial membrane systems resembling the lipid composition of the natural thylakoid membrane. *Biochim. Biophys. Acta* 1768 67–75. 10.1016/j.bbamem.2006.06.006 16843433

[B54] Goss R. Lepetit B. (2015). Biodiversity of NPQ. *J. Plant Physiol.* 172 13–32. 10.1016/j.jplph.2014.03.004 24854581

[B55] Goss R. Lohr M. Latowski D. Grzyb J. Vieler A. Wilhelm C. (2005). Role of hexagonal structure-forming lipids in diadinoxanthin and violaxanthin solubilization and de-epoxidation. *Biochemistry* 44 4028–4036. 10.1021/bi047464k 15751979

[B56] Goss R. Nerlich J. Lepetit B. Schaller S. Vieler A. Wilhelm C. (2009). The lipid dependence of diadinoxanthin de-epoxidation presents new evidence for a macrodomain organization of the diatom thylakoid membrane. *J. Plant Physiol.* 166 1839–1854. 10.1016/j.jplph.2009.05.017 19604599

[B57] Goss R. Pinto E. A. Wilhelm C. Richter M. (2006). The importance of a highly active and ΔpH-regulated diatoxanthin epoxidase for the regulation of the PS II antenna function in diadinoxanthin cycle containing algae. *J. Plant Physiol.* 163 1008–1021. 10.1016/j.jplph.2005.09.008 16971213

[B58] Goss R. Wilhelm C. (2009). “Lipids in algae, lichens and mosses,” in *Lipids in Photosynthesis: Essential and Regulatory Functions*, eds Wada H. Murata N. Govindjee M. (Berlin: Springer), 119–120.

[B59] Gounaris K. Brain A. P. R. Quinn P. J. Williams W. P. (1984). Structural reorganization of chloroplast thylakoid membranes in response to heat stress. *Biochim. Biophys. Acta* 766 198–208. 10.1016/0005-2728(84)90232-9

[B60] Grouneva I. Jakob T. Wilhelm C. Goss R. (2006). Influence of ascorbate and pH on the activity of the diatom xanthophyll cycle-enzyme diadinoxanthin de-epoxidase. *Physiol. Plant.* 126 205–211. 10.1111/j.1399-3054.2006.00613.x

[B61] Grudzinski W. Nierzwicki L. Welc R. Reszczynska E. Luchowski R. Czub J. (2017). Localization and orientation of xanthophylls in a lipid bilayer. *Sci. Rep.* 7:9619. 10.1038/s41598-017-10183-7 PMC5575131 28852075

[B62] Gruszecki W. I. Strzalka K. (1991). Does the xanthophyll cycle take part in the regulation of fluidity of the thylakoid membrane? *Biochim. Biophys. Acta* 1060 310–314. 10.1016/s0005-2728(05)80322-6

[B63] Gruszecki W. I. Strzalka K. (2005). Carotenoids as modulators of lipid membrane physical properties. *Biochim. Biophys. Acta* 1740 108–115. 10.1016/j.bbadis.2004.11.015 15949676

[B64] Grzyb J. Latowski D. Strzalka K. (2006). Lipocalins - a family portrait. *J. Plant Physiol.* 163 895–915. 10.1016/j.jplph.2005.12.007 16504339

[B65] Gundermann K. Büchel C. (2008). The fluorescence yield of the trimeric fucoxanthin-chlorophyll-protein FCPa in the diatom *Cyclotella meneghiniana* is dependent on the amount of bound diatoxanthin. *Photosynth. Res.* 95 229–235. 10.1007/s11120-007-9262-1 17912602

[B66] Gundermann K. Wagner V. Mittag M. Büchel C. (2019). Fucoxanthin-chlorophyll protein complexes of the centric diatom *Cyclotella meneghiniana* differ in Lhcx1 and Lhcx6_1 content. *Plant Physiol.* 179 1779–1795. 10.1104/pp.18.01363 30733257 PMC6446762

[B67] Guschina I. A. Harwood J. L. (2006). Lipids and lipid metabolism in eukaryotic algae. *Prog. Lipid Res.* 45, 160–186. 10.1016/j.plipres.2006.01.001 16492482

[B68] Guskov A. Kern J. Saenger W. (2009). Cyanobacterial photosystem II at 2.9-A resolution and the role of quinones, lipids, channels and chloride. *Nat. Struct. Mol. Biol.* 16 334–342. 10.1038/nsmb.1559 19219048

[B69] Hager A. (1957). Über den Einfluss klimatischer Faktoren auf den Blattfarbstoffgehalt höherer Pflanzen. *Planta* 49 524–560. 10.1007/bf01917726

[B70] Hager A. (1967a). Untersuchungen über die lichtinduzierten, reversiblen Xanthophyllumwandlungen an *Chlorella* und *Spinacia oleracea*. *Planta* 74 148–172. 10.1007/bf00388326 24549888

[B71] Hager A. (1967b). Untersuchungen üben die Rückreaktionen im Xanthophyll-Cyclus bei *Chlorella*, *Spinacia* und *Taxus. Planta* 76, 138–148. 10.1007/BF00385460 24549422

[B72] Hager A. (1969). Lichtbedingte pH-Erniedrigung in einem *Chloroplasten-Kompartiment* als Ursache der enzymatischen Violaxanthin-Zeaxanthin-Umwandlung: Beziehungen zur Photophosphorylierung. *Planta* 89 224–243. 10.1007/bf00385028 24504466

[B73] Hager A. Holocher K. (1994). Localization of the xanthophyll-cycle enzyme violaxanthin de-epoxidase within the thylakoid lumen and abolition of its mobility by a (light-dependent) pH decrease. *Planta* 192 581–589.

[B74] Hager A. Stransky H. (1970). Das Carotinoidmuster und die Verbreitung des lichtinduzierten Xanthophyllcyclus in verschiedenen Algenklassen. *Arch. Mikrobiol.* 73 77–89. 10.1007/bf00409954 5484315

[B75] Hallin E. I. Guo K. Akerlund H. E. (2015). Violaxanthin de-epoxidase disulphides and their role in activity and thermal stability. *Photosynth Res.* 124 191–198. 10.1007/s11120-015-0118-119 25764016 PMC4412432

[B76] Hallin E. I. Hasan M. Guo K. Akerlund H.-E. (2016). Molecular studies on structural changes and oligomerisation of violaxanthin deepoxidase associated with the pH-dependent activation. *Photosynth. Res.* 129 29–41. 10.1007/s11120-016-0261-y 27116125

[B77] Haranczyk H. Strzalka K. Dietrich W. Blicharski J. S. (1995). ^13^P-NMR observation of the temperature and glycerol induced non-lamellar phase formation in wheat thylakoid membranes. *J. Biol. Phys.* 21 125–139. 10.1007/bf00705595

[B78] Härtel H. Benning C. (2000). Can digalactosyldiacylglycerol substitute for phosphatidylcholine upon phosphate deprivation in leaves and roots of Arabidopsis? *Biochem. Soc. Trans.* 28 729–732. 10.1042/bst0280729 11171187

[B79] Harwood J. L. Guschina I. A. (2009). The versatility of algae and their lipid metabolism. *Biochimie* 91 679–684. 10.1016/j.biochi.2008.11.004 19063932

[B80] Havaux M. (1998). Carotenoids as membrane stabilizers in chloroplasts. *Trends Plant Sci.* 3 147–151. 10.1016/s1360-1385(98)01200-x

[B81] Havaux M. Dall’Osto L. Bassi R. (2007). Zeaxanthin has enhanced antioxidant capacity with respect to all other xanthophylls in *Arabidopsis* leaves and functions independent of binding to PSII antennae. *Plant Physiol.* 145 1506–1520. 10.1104/pp.107.108480 17932304 PMC2151694

[B82] Havaux M. Niyogi K. K. (1999). The violaxanthin cycle protects plants from photooxidative damage by more than one mechanism. *Proc. Natl. Acad. Sci. U.S.A.* 96 8762–8767. 10.1073/pnas.96.15.8762 10411949 PMC17590

[B83] Heemskerk J. W. M. Wintermans J. F. G. M. Joyard J. Block M. A. Dorne A. J. Douce R. (1986). Localization of galactolipid–galactolipid galactosyltransferase and acyltransferase in outer envelope membrane of spinach chloroplasts. *Biochim. Biophys. Acta* 877 281–289. 10.1016/0005-2760(86)90305-x

[B84] Heinz E. Roughan P. G. (1983). Similarities and differences in lipid metabolism of chloroplasts isolated from 18:3 and 16:3 plants. *Plant Physiol.* 72 273–279. 10.1104/pp.72.2.273 16662992 PMC1066223

[B85] Hieber A. D. Bugos R. C. Yamamoto H. Y. (2000). Plant lipocalins: violaxanthin de-epoxidase and zeaxanthin epoxidase. *Biochim. Biophys. Acta* 1482 84–91. 10.1016/s0167-4838(00)00141-2 11058750

[B86] Higashi Y. Okazaki Y. Myouga F. Shinozaki K. Saito K. (2015). Landscape of the lipidome and transcriptome under heat stress in *Arabidopsis thaliana*. *Sci. Rep.* 5:10533. 10.1038/srep10533 PMC4444972 26013835

[B87] Higashi Y. Okazaki Y. Takano K. Myouga F. Shinozaki K. Knoch E. (2018). A lipase gene, HEAT INDUCIBLE LIPASE1, is involved in remodeling chloroplastic monogalactosyldiacylglycerol by liberatingα-linolenic acid in *Arabidopsis* leaves under heat stress. *Plant Cell* 30 1887–1905. 10.1105/tpc.18.00347 29967047 PMC6139690

[B88] Higashi Y. Saito K. (2019). Lipidomic studies of membrane glycerolipids in plant leaves under heat stress. *Prog. Lipid Res.* 75:100990 10.1016/j.plipres.2019.100990 31442527

[B89] Hölzl G. Dörmann P. (2019). Chloroplast lipids and their biosynthesis. *Annu. Rev. Plant Biol.* 70 51–81. 10.1146/annurev-arplant-050718-100202 30786236

[B90] Holzwarth A. R. Miloslavina Y. Nilkens M. Jahns P. (2009). Identification of two quenching sites active in the regulation of photosynthetic light-harvesting studied by time-resolved fluorescence. *Chem. Phys. Lett.* 483 262–267. 10.1016/j.cplett.2009.10.085

[B91] Horton P. (2014). “Developments in research on non-photochemical fluorescence quenching: emergence of key ideas, theories and experimental approaches,” in *Non-Photochemical Quenching And Energy Dissipation In Plants, Algae And Cyanobacteria*, eds Demmig-Adams B. Garab G. Adams W. W. (Dordrecht: Springer), 73–95. 10.1007/978-94-017-9032-1_3

[B92] Huang Y. Gui S. (2018). Factors affecting the structure of lyotropic liquid crystals and the correlation between structure and drug diffusion. *RSC Adv.* 8 6978–6987. 10.1039/c7ra12008g PMC9078419 35540315

[B93] Jahns P. Holzwarth A. R. (2012). The role of the xanthophyll cycle and of lutein in photoprotection of photosystem II. *Biochim. Biophys. Acta* 1817 182–193. 10.1016/j.bbabio.2011.04.012 21565154

[B94] Jahns P. Latowski D. Strzalka K. (2009). Mechanism and regulation of the violaxanthin cycle: the role of antenna proteins and membrane lipids. *Biochim. Biophys. Acta* 1787 3–14. 10.1016/j.bbabio.2008.09.013 18976630

[B95] Jahns P. Wehner A. Paulsen H. Hobe S. (2001). De-epoxidation of violaxanthin after reconstitution into different carotenoid binding sites of light-harvesting complex II. *J. Biol. Chem.* 276 22154–22159. 10.1074/jbc.m102147200 11301335

[B96] Jakob T. Goss R. Wilhelm C. (2001). Unusual pH-dependence of diadinoxanthin de-epoxidase activation causes chlororespiratory induced accumulation of diatoxanthin in the diatom *Phaeodactylum tricornutum*. *J. Plant Physiol.* 158 383–390. 10.1078/0176-1617-00288

[B97] Janero D. R. Barrnett R. (1982). Isolation and characterization of an ether-linked homoserine lipid from the thylakoid membrane of *Chlamydomonas reinhardtii* 137+. *J. Lipid Res.* 23 307–316. 7077145

[B98] Jarvis P. Dörmann P. Chory J. (2000). Galactolipid deficiency and abnormal chloroplast development in the *Arabidopsis* MGD synthase 1 mutant. *Proc. Natl. Acad. Sci. U.S.A.* 97 8175–8179. 10.1073/pnas.100132197 10869420 PMC16689

[B99] Johnson M. P. Havaux M. Triantaphylides C. Ksas B. Pascal A. A. Robert B. (2007). Elevated zeaxanthin bound to oligomeric LHCII enhances the resistance of Arabidopsis to photooxidative stress by a lipid-protective, antioxidant mechanism. *J. Biol. Chem.* 282 22605–22618. 10.1074/jbc.m702831200 17553786

[B100] Jouhet J. (2013). Importance of the hexagonal lipid phase in biological membrane organization. *Front. Plant Sci.* 4:494 10.3389/fpls.2013.00494 PMC3848315 24348497

[B101] Jouhet J. Maréchal E. Baldan B. Bligny R. Joyard J. Block M. A. (2004). Phosphate deprivation induces transfer of DGDG galactolipid from chloroplast to mitochondria. *J. Cell. Biol.* 167 863–874. 10.1083/jcb.200407022 15569715 PMC2172463

[B102] Kansy M. Wilhelm C. Goss R. (2014). Influence of thylakoid membrane lipids on the structure and function of the plant photosystem II core complex. *Planta* 240 781–796. 10.1007/s00425-014-2130-2 25063517

[B103] Killian J. A. (1998). Hydrophobic mismatch between proteins and lipids in membranes. *Biochim. Biophys. Acta* 1376 401–416. 10.1016/s0304-4157(98)00017-3 9805000

[B104] Kirchhoff H. (2014). Diffusion of molecules and macromolecules in thylakoid membranes. *Biochim. Biophys. Acta* 1837 495–502. 10.1016/j.bbabio.2013.11.003 24246635

[B105] Kirchhoff H. (2018). Structure-function relationships in photosynthetic membranes: challenges and emerging fields. *Plant Sci.* 266, 76–82. 10.1016/j.plantsci.2017.09.021 29241569

[B106] Kirchhoff H. Mukherjee U. Galla H.-J. (2002). Molecular architecture of the thylakoid membrane: lipid diffusion space for plastoquinone. *Biochemistry* 41 4872–4882. 10.1021/bi011650y 11939782

[B107] Kobayashi K. Endo K. Wada H. (2016). “Roles of lipids in photosynthesis,” in *Lipids in Plant and Algae Development*, eds Nakamura Y. Li-Beisson Y. (Berlin: Springer), 21–49. 10.1007/978-3-319-25979-6_2 27023230

[B108] Kobayashi K. Kondo M. Fukuda H. Nishimura M. Ohta H. (2007). Galactolipid synthesis in chloroplast inner envelope is essential for proper thylakoid biogenesis, photosynthesis, and embryogenesis. *Proc. Natl. Acad. Sci. U.S.A.* 104 17216–17221. 10.1073/pnas.0704680104 17940034 PMC2040463

[B109] Kotakis C. Akhtar P. Zsiros O. Garab G. Lambrev P. H. (2018). Increased thermal stability of photosystem II and the macro-organization of thylakoid membranes, induced by co-solutes, associated with changes in the lipid-phase behaviour of thylakoid membranes. *Photosynthetica* 56 254–264. 10.1007/s11099-018-0782-z

[B110] Krauss S. (2001). “Mitochondria: structure and role in respiration,” in *Encyclopedia of Life Sciences*, (New York, NY: Nature Publishing Group), 1–6.

[B111] Krumova S. B. Dijkema C. de Waard P. van As H. Garab G. van Amerongen H. (2008). Phase behavior of phosphatidylglycerol in spinach thylakoid membranes as revealed by ^31^P-NMR. *Biochim. Biophys. Acta* 1778 997–1003. 10.1016/j.bbamem.2008.01.004 18230332

[B112] Kumari P. Kumar M. Reddy C. R. K. Jha B. (2013). “Algal lipids, fatty acids and sterols,” in *Functional Ingredients From Algae For Foods And Nutraceuticals*, ed. Dominguez H. (Sawston: Woodhead Publishing Ltd), 87–134. 10.1533/9780857098689.1.87

[B113] Latowski D. Akerlund H.-E. Strzalka K. (2004). Violaxanthin de-epoxidase, the xanthophyll cycle enzyme, requires lipid inverted hexagonal structures for its activity. *Biochemistry* 43 4417–4420. 10.1021/bi049652g 15078086

[B114] Latowski D. Kostecka A. Strzalka K. (2000). Effect of monogalactosyldiacylglycerol and other thylakoid lipids on violaxanthin de-epoxidation in liposomes. *Biochem. Soc. Trans.* 28 810–812. 10.1042/bst0280810 11171216

[B115] Latowski D. Kruk J. Burda K. Skrzynecka-Jaskier M. Kostecka-Gugala A. Strzalka K. (2002). Kinetics of violaxanthin de-epoxidation by violaxanthin de-epoxidase, a xanthophyll cycle enzyme, is regulated by membrane fluidity in model lipid bilayers. *Eur. J. Biochem.* 269 4656–4665. 10.1046/j.1432-1033.2002.03166.x 12230579

[B116] Lavaud J. Lepetit B. (2013). An explanation for the inter-species variability of the photo-protective non-photochemical chlorophyll fluorescence quenching in diatoms. *Biochim. Biophys. Acta* 1827 294–302. 10.1016/j.bbabio.2012.11.012 23201475

[B117] Lavaud J. Rousseau B. Etienne A. (2003). Enrichment of the light-harvesting complex in diadinoxanthin and implications for the nonphotochemical fluorescence quenching in diatoms. *Biochemistry* 42 5802–5808. 10.1021/bi027112i 12741838

[B118] Lee A. L.-C. Thornber J. P. (1995). Analysis of the pigment stoichiometry of pigment-protein complexes from barley (*Hordeum vulgare*). *Plant Physiol.* 107 565–574. 10.1104/pp.107.2.565 7724673 PMC157160

[B119] Leonelli L. Brooks M. D. Niyogi K. K. (2017). Engineering the lutein epoxide cycle into *Arabidopsis thaliana*. *Proc. Natl. Acad. Sci. U.S.A.* 114 E7002–E7008. 10.1073/pnas.1704373114 28760990 PMC5565435

[B120] Lepetit B. Goss R. Jakob T. Wilhelm C. (2012). Molecular dynamics of the diatom thylakoid membrane under different light conditions. *Photosynth. Res.* 111 245–257. 10.1007/s11120-011-9633-5 21327535

[B121] Lepetit B. Sturm S. Rogato A. Gruber A. Sachse M. Falciatore A. (2013). High light acclimation in the secondary plastids containing diatom *Phaeodactylum tricornutum* is triggered by the redox state of the plastoquinone pool. *Plant Physiol.* 161 853–865. 10.1104/pp.112.207811 23209128 PMC3561024

[B122] Lepetit B. Volke D. Gilbert M. Wilhelm C. Goss R. (2010). Evidence for the existence of one antenna-associated, lipid-dissolved, and two protein-bound pools of diadinoxanthin cycle pigments in diatoms. *Plant Physiol.* 154 1905–1920. 10.1104/pp.110.166454 20935178 PMC2996015

[B123] Lepetit B. Volke D. Szabo M. Hoffmann R. Garab G. Wilhelm C. (2008). “The oligomeric antenna of the diatom *P. tricornutum* - localization of diadinoxanthin cycle pigments,” in *Photosynthesis. Energy from the sun*, eds Allen J. F. Gantt E. Golbeck J. H. Osmond B. (Berlin: Springer), 277–280.

[B124] Li M. Welti R. Wang X. (2006). Quantitative profiling of Arabidopsis polar glycerolipids in response to phosphorus starvation. Roles of phospholipases D zeta1 and D zeta 2 in phosphatidylcholine hydrolysis and digalactosyldiacylglycerol accumulation in phosphorus-starved plants. *Plant Physiol.* 142 750–761. 10.1104/pp.106.085647 16891548 PMC1586058

[B125] Li Q. Zheng Q. Shen W. Cram D. Fowler D. B. Wei Y. (2015). Understanding the biochemical basis of temperature induced lipid pathway adjustments in plants. *Plant Cell* 27 86–103. 10.1105/tpc.114.134338 25564555 PMC4330585

[B126] Li X. Björkman O. Shih C. Grossman A. R. Rosenquist M. Jansson S. (2000). A pigment-binding protein essential for regulation of photosynthetic light harvesting. *Nature* 403 391–395. 10.1038/35000131 10667783

[B127] Li Z. Peers G. Dent R. M. Bai Y. Yang S. Y. Apel W. (2016). Evolution of an atypical de-epoxidase for photoprotection in the green lineage. *Nat. Plants* 2:16140. 10.1038/nplants.2016.140 PMC5021192 27618685

[B128] Li-Beisson Y. Nakamura Y. Harwood J. (2016). Lipids: from chemical structures, biosynthesis, and analyses to industrial applications. *Subcell. Biochem.* 86 1–21. 27023229 10.1007/978-3-319-25979-6_1

[B129] Liu Z. Yan H. Wang K. Kuang T. Zhang J. Gui L. (2004) crystal structure of spinach major light-harvesting complex at 2.72 A resolution. *Nature* 428, 287–292. 10.1038/nature02373 15029188

[B130] Lohr M. Wilhelm C. (1998). Algae displaying the diadinoxanthin cycle also possess the violaxanthin cycle. *Proc. Natl. Acad. Sci. U.S.A.* 96 8784–8789. 10.1073/pnas.96.15.8784 PMC17594 10411953

[B131] Lohr M. Wilhelm C. (2001). Xanthophyll synthesis in diatoms: quantification of putative intermediates and comparison of pigment conversion kinetics with rate constants derived from a model. *Planta* 212 382–391. 10.1007/s004250000403 11289603

[B132] Macko S. Wehner A. Jahns P. (2002). Comparison of violaxanthin de-epoxidation from the stroma and lumen sides of isolated thylakoid membranes from Arabidopsis: Implications for the mechanism of de-epoxidation. *Planta* 216 309–314. 10.1007/s00425-002-0848-8 12447545

[B133] Matsuda O. Sakamoto H. Hashimoto T. Iba K. (2005). A temperature-sensitive mechanism that regulates post-translational stability of a plastidial omega-3 fatty acid desaturase (FAD8) in Arabidopsis leaf tissues. *J. Biol. Chem.* 280 3597–3604. 10.1074/jbc.m407226200 15545277

[B134] Mewes H. Richter M. (2002). Supplementary Ultraviolet-B radiation induces a rapid reversal of the diadinoxanthin cycle in the strong light-exposed diatom *Phaeodactylum tricornutum*. *Plant Physiol.* 130 1527–1535. 10.1104/pp.006775 12428017 PMC166671

[B135] Miloslavina Y. Grouneva I. Lambrev P. H. Lepetit B. Goss R. Wilhelm C. (2009). Ultra-fast fluorescence study on the location and mechanism of non-photochemical quenching in diatoms. *Biochim. Biophys. Acta* 1787 1189–1197. 10.1016/j.bbabio.2009.05.012 19486881

[B136] Moellering E. R. Benning C. (2011). Galactoglycerolipid metabolism under stress: a time for remodeling. *Trends Plant Sci.* 16 98–107. 10.1016/j.tplants.2010.11.004 21145779

[B137] Moellering E. R. Muthan B. Benning C. (2010). Freezing tolerance in plants requires lipid remodeling at the outer chloroplast membrane. *Science* 330 226–228. 10.1126/science.1191803 20798281

[B138] Mongrand S. Bessoule J.-J. Cabantous F. Cassagne C. (1998). The C16:3/C18:3 fatty acid balance in photosynthetic tissues from 468 plant species. *Phytochemistry* 49 1049–1064. 10.1016/s0031-9422(98)00243-x

[B139] Mongrand S. Stanislas T. Bayer E. M. F. Lherminier J. Simon-Plas F. (2010). Membrane rafts in plant cells. *Trends Plant Sci.* 15 656–663. 10.1016/j.tplants.2010.09.003 20934367

[B140] Montsant A. Allen A. E. Coesel S. De Martino A. Falciatore A. Mangogna M. (2007). Identification and comparative genomic analysis of signaling and regulatory components in the diatom *Thalassiosira pseudonana*. *J. Phycol.* 43 585–604.

[B141] Morosinotto T. Caffarri S. Dall’Osto L. Bassi R. (2003). Mechanistic aspects of the xanthophyll dynamics in higher plant thylakoids. *Physiol. Plant.* 119 347–354. 10.1034/j.1399-3054.2003.00213.x

[B142] Mène-Saffrané L. Dubugnon L. Chételat A. Stolz S. Gouhier-Darimont C. Farmer E. (2009). Nonenzymatic oxidation of trienoic fatty acids contributes to reactive oxygen species management in *Arabidopsis*. *J. Biol. Chem.* 284 1702–1708. 10.1074/jbc.m807114200 18996838

[B143] Nawrocki W. J. Liu X. Croce R. (2019). *Chlamydomonas reinhardtii* exhibits de facto constitutive NPQ capacity in physiologically relevant conditions. *Plant Physiol.* 182 472–479. 10.1104/pp.19.00658 31653716 PMC6945880

[B144] Nicholson G. L. (2014). The fluid-mosaic model of membrane structure: Still relevant to understanding the structure, function and dynamics of biological membranes after more than 40 years. *Biochim. Biophys. Acta* 1838 1451–1466. 10.1016/j.bbamem.2013.10.019 24189436

[B145] Nicol L. Nawrocki W. J. Croce R. (2019). Disentangling the sites of non-photochemical quenching in vascular plants. *Nature Plants* 5 1177–1183. 10.1038/s41477-019-0526-5 31659240 PMC6861128

[B146] Niyogi K. K. Truong T. B. (2013). Evolution of flexible non-photochemical quenching mechanisms that regulate light harvesting in oxygenic photosynthesis. *Curr. Opin. Plant Biol.* 16 307–314. 10.1016/j.pbi.2013.03.011 23583332

[B147] Ohlrogge J. B. Kuhn D. N. Stumpf P. K. (1979). Subcellular localization of acyl carrier protein in leaf protoplasts of *Spinacia oleracea*. *Proc. Natl. Acad. Sci. U.S.A.* 76 1194–1198. 10.1073/pnas.76.3.1194 286305 PMC383216

[B148] Okazaki Y. Otsuki H. Narisawa T. Kobayashi M. Sawai S. Kamide Y. (2013). A new class of plant lipid is essential for protection against phosphorus depletion. *Nat. Commun.* 4:1510. 10.1038/ncomms2512 PMC3586718 23443538

[B149] Peers G. Truong T. B. Ostendorf E. Busch A. Elrad D. Grossman A. R. (2009). An ancient light-harvesting protein is critical for the regulation of algal photosynthesis. *Nature* 462 518–521. 10.1038/nature08587 19940928

[B150] Pfündel E. E. Renganathan M. Gilmore A. M. Yamamoto H. Y. Dilley R. A. (1994). Intrathylakoid pH in isolated Pea chloroplasts as probed by violaxanthin deepoxidation. *Plant Physiol.* 106 1647–1658. 10.1104/pp.106.4.1647 12232439 PMC159709

[B151] Rabinowitch H. D. Budowski P. Kedar N. (1975). Carotenoids and epoxide cycles in mature-green tomatoes. *Planta* 122 91–97. 10.1007/bf00385408 24435925

[B152] Ramani B. Reeck T. Debez A. Stelzer R. Huchzermeyer B. Schmidt A. (2006). *Aster tripolium* L. and *Sesuvium portulacastrum* L.: two halophytes, two strategies to survive in saline habitats. *Plant Physiol. Biochem.* 44 395–408. 10.1016/j.plaphy.2006.06.007 16806957

[B153] Roughan G. Slack R. (1984). Glycerolipid synthesis in leaves. *Trends Biochem. Sci.* 9 383–386. 10.1016/0968-0004(84)90220-2

[B154] Ruban A. V. (2016). Nonphotochemical fluorescence quenching: mechanism and effectiveness in protecting plants from photodamage. *Plant Physiol.* 170 1903–1916. 10.1104/pp.15.01935 26864015 PMC4825125

[B155] Ruban A. V. Lee P. J. Wentworth M. Young A. J. Horton P. (1999). Determination of the stoichiometry and strength of binding of xanthophylls to the photosystem II light harvesting complexes. *J. Biol. Chem.* 274 10458–10465. 10.1074/jbc.274.15.10458 10187836

[B156] Ruban A. V. Phillip D. Young A. J. Horton P. (1997). Carotenoid-dependent oligomerization of the major chlorophyll a/b light harvesting complex of photosystem II of plants. *Biochemistry* 36 7855–7859. 10.1021/bi9630725 9201929

[B157] Ruban A. V. Rees D. Pascal A. A. Horton P. (1992). Mechanism of ΔpH dependent dissipation of absorbed excitation energy by photosynthetic membranes. II. The relation-ship between LHCII aggregation in vitro and qE in isolated thylakoids. *Biochim. Biophys. Acta* 1102 39–44. 10.1016/0005-2728(92)90062-7

[B158] Ruban A. V. Young A. J. Pascal A. A. Horton P. (1994). The effects of illumination on the xanthophyll composition of the photosystem II light-harvesting complexes of spinach thylakoid membranes. *Plant Physiol.* 104 227–234. 10.1104/pp.104.1.227 12232075 PMC159181

[B159] Sacharz J. Giovagnetti V. Ungerer P. Mastroianni G. Ruban A. V. (2017). The xanthophyll cycle affects reversible interactions between PsbS and light-harvesting complex II to control non-photochemical quenching. *Nature Plants* 3:16225. 10.1038/nplants.2016.225 28134919

[B160] Saga G. Giorgetti A. Fufezan C. Giacometti G. M. Bassi R. Morosinotto T. (2010). Mutation analysis of violaxanthin de-epoxidase identifies substrate-binding sites and residues involved in catalysis. *J. Biol. Chem.* 285 23763–23770. 10.1074/jbc.m110.115097 20507981 PMC2911307

[B161] Saphozhnikov D. J. Krasovskaya T. A. Mayevskaya A. N. (1957). Changes observed in the relation between the main carotenoids in the plastids of green leaves exposed to light. *Dokl. Akad. Nauk. SSSR 1* 13 465–467.

[B162] Schaller S. Latowski D. Jemiola-Rzeminska M. Quaas T. Wilhelm C. Strzalka K. (2012a). The investigation of violaxanthin de-epoxidation in the primitive green alga *Mantoniella squamata* (Prasinophyceae) indicates mechanistic differences in xanthophyll conversion to higher plants. *Phycologia* 51 359–370. 10.2216/11-127.1

[B163] Schaller S. Wilhelm C. Strzalka K. Goss R. (2012b). Investigating the interaction between the violaxanthin cycle enzyme zeaxanthin epoxidase and the thylakoid membrane. *J. Photochem. Photobiol. B: Biol.* 2012 119–125. 10.1016/j.jphotobiol.2012.05.019 22705077

[B164] Schaller S. Latowski D. Jemiola-Rzeminska M. Wilhelm C. Strzalka K. Goss R. (2010). The main thylakoid membrane lipid monogalactosyldiacylglycerol (MGDG) promotes the de-epoxidation of violaxanthin associated with the light-harvesting complex of photosystem II (LHCII). *Biochim. Biophys. Acta* 2 414–424. 10.1016/j.bbabio.2009.12.011 20035710

[B165] Schaller S. Latowski D. Jemioła-Rzemioska M. Dawood A. Wilhelm C. Strzałka K. (2011). Regulation of LHCII aggregation by different thylakoid membrane lipids. *Biochim. Biophys. Acta* 1807 326–335. 10.1016/j.bbabio.2010.12.017 21215252

[B166] Schaller S. Richter K. Wilhelm C. Goss R. (2014). Influence of pH, Mg^2 +^, and lipid composition on the aggregation state of the diatom FCP in comparison to the LHCII of vascular plants. *Photosynth. Res.* 119 305–317. 10.1007/s11120-013-9951-x 24197266

[B167] Schaller-Laudel S. Latowski D. Jemioła-Rzeminska M. Strzałka K. Daum S. Bacia K. (2017). Influence of thylakoid membrane lipids on the structure of aggregated light-harvesting complexes of the diatom *Thalassiosira pseudonana* and the green alga *Mantoniella squamata*. *Physiol. Plant.* 160 339–358. 10.1111/ppl.12565 28317130

[B168] Schaller-Laudel S. Volke D. Redlich M. Kansy M. Hoffmann R. Wilhelm C. (2015). The diadinoxanthin diatoxanthin cycle induces structural rearrangements of the isolated FCP antenna complexes of the pennate diatom *Phaeodactylum tricornutum*. *Plant Physiol. Biochem.* 96 364–376. 10.1016/j.plaphy.2015.09.002 26368016

[B169] Schmid-Siegert E. Stepushenko O. Glauser G. Farmer E. (2016). Membranes as structural antioxidants - recycling of malondialdehyde to its source in oxidation-sensitive chloroplast fatty acids. *J. Biol. Chem.* 291 13005–13013. 10.1074/jbc.m116.729921 27143359 PMC4933218

[B170] Schumann A. Goss R. Jakob T. Wilhelm C. (2007). Investigation of the quenching efficiency of diatoxanthin in cells of *Phaeodactylum tricornutum* (Bacillariophyceae) with different pool sizes of xanthophyll cycle pigments. *Phycologia* 46 113–117. 10.2216/06-30.1

[B171] Seiwert D. Witt H. Janshoff A. Paulsen H. (2017). The non-bilayer lipid MGDG stabilizes the major light-harvesting complex (LHCII) against unfolding. *Sci. Rep.* 7:5158. 10.1038/s41598-017-05328-7 PMC5505961 28698661

[B172] Selstam E. (1998). “Development of thylakoid membranes with respect to lipids,” in *Lipids in Photosynthesis: Structure, Function And Genetics*, eds Siegenthaler P. A. Murata N. (Dordrecht: Kluwer Academic Publishers), 209–224. 10.1007/0-306-48087-5_11

[B173] Shimojima M. (2011). Biosynthesis and functions of the plant sulfolipid. *Progr. Lipid Res.* 50 234–239. 10.1016/j.plipres.2011.02.003 21371504

[B174] Shipley G. G. Green J. P. Nichols B. W. (1973). The phase behaviour of monogalactosyl, digalactosyl, and sulphoquinovosyl diglycerides. *Biochim. Biophys. Acta* 311 531–544. 10.1016/0005-2736(73)90128-4 4738152

[B175] Simidjiev I. Barzda V. Mustardy L. Garab G. (1998). Role of thylakoid lipids in the structural flexibility of lamellar aggregates of the isolated light-harvesting chlorophyll a/b complex of photosystem II. *Biochemistry* 37 4169–4173. 10.1021/bi972726m 9521738

[B176] Simidjiev I. Stoylova S. Amenitsch H. Javorfi T. Mustardy L. Laggner P. (2000). Self-assembly of large, ordered lamellae from non-bilayer lipids and integral membrane proteins in vitro. *Proc. Natl. Acad. Sci. U.S.A.* 97 1473–1476. 10.1073/pnas.97.4.1473 10677486 PMC26458

[B177] Simionato D. Basso S. Zaffagnini M. Lana T. Marzotto F. Trost P. (2015). Protein redox regulation in the thylakoid lumen: the importance of disulfide bonds for violaxanthin deepoxidase. *FEBS Lett.* 589 919–923. 10.1016/j.febslet.2015.02.033 25747136

[B178] Simon-Plas F. Perraki A. Bayer E. Gerbot-Pissot P. Mongrand S. (2011). An update on plant membrane rafts. *Curr. Opin. Plant Biol.* 14 642–649. 10.1016/j.pbi.2011.08.003 21903451

[B179] Singer S. J. Nicholson G. L. (1972). The fluid mosaic model of the structure of cell membranes. *Science* 175 720–731. 10.1126/science.175.4023.720 4333397

[B180] Stransky H. Hager A. (1970). Das Carotinoidmuster und die Verbreitung des lichtinduzierten Xanthophyllzyklus in verschiedenen Algenklassen. II: Xanthophyceae. *Arch. Mikrobiol.* 71 164–190. 10.1007/bf00417740 5448506

[B181] Taddei L. Chukhutsina V. U. Lepetit B. Stella G. R. Bassi R. van Amerongen H. (2018). Dynamic changes between two LHCX-related energy quenching sites control diatom photoacclimation. *Plant Physiol.* 177 953–965. 10.1104/pp.18.00448 29773581 PMC6053010

[B182] Taddei L. Stella G. R. Rogato A. Bailleul B. Fortunato A. E. Annunziata R. (2016). Multisignal control of expression of the LHCX protein family in the marine diatom *Phaeodactylum tricornutum*. *J. Exp. Bot.* 67 3939–3951. 10.1093/jxb/erw198 27225826 PMC4915529

[B183] Tardy F. Havaux M. (1997). Thylakoid membrane fluidity and thermostability during the operation of the xanthophyll cycle in higher plant chloroplasts. *Biochim. Biophys. Acta* 1330 179–193. 10.1016/s0005-2736(97)00168-5 9408171

[B184] Thomas P. G. Brain A. P. R. Quinn P. J. Williams W. P. (1985). Low pH and phospholipase A2 treatment induce the phase separation of non-bilayer lipids within pea chloroplast membranes. *FEBS Letts.* 183 161–166. 10.1016/0014-5793(85)80976-5

[B185] Triantaphylidès C. Havaux M. (2009). Singlet oxygen in plants: production, detoxification and signaling. *Trends Plant Sci.* 14 219–228. 10.1016/j.tplants.2009.01.008 19303348

[B186] van Besouw A. Wintermans J. F. (1978). Galactolipid formation in chloroplast envelopes. I. Evidence for two mechanisms in galactosylation. *Biochim. Biophys. Acta* 529 44–53. 10.1016/0005-2760(78)90102-9 638180

[B187] van den Brink-van der Laan E. Killian J. A. de Kruijff B. (2004). Non-bilayer lipids affect peripheral and integral membrane proteins via changes in the lateral pressure profile. *Biochim. Biophys. Acta* 1666 275–288. 10.1016/j.bbamem.2004.06.010 15519321

[B188] van Eerden F. J. de Jong D. H. de Vries A. H. Wassenaar T. A. Marrink S. J. (2015). Characterization of thylakoid lipid membranes from cyanobacteria and higher plants by molecular dynamics simulations. *Biochim. Biophys. Acta* 1848 1319–1330. 10.1016/j.bbamem.2015.02.025 25749153

[B189] van Eerden F. J. Melo M. N. Frederix P. W. J. M. Marrink S. J. (2017). Prediction of thylakoid lipid binding sites on Photosystem II. *Biophys. J.* 113 2669–2681. 10.1016/j.bpj.2017.09.039 29262360 PMC5770566

[B190] van Mooy B. A. S. Rocap G. Fredricks H. F. Evans C. T. Devol A. H. (2006). Sulfolipids dramatically decrease phosphorus demand by picocyanobacteria in oligotrophic marine environments. *Proc. Natl. Acad. Sci. U.S.A.* 103 8607–8612. 10.1073/pnas.0600540103 16731626 PMC1482627

[B191] Vidal-Meireles A. Toth D. Kovacz L. Neupert J. Toth S. Z. (2020). Ascorbate deficiency does not limit non-photochemical quenching in *Chlamydomonas reinhardtii*. *Plant Physiol.* 182:E00916.2019 10.1104/pp.19.00916 PMC6945847 31662419

[B192] Vieler A. Wilhelm C. Goss R. Süss R. Schiller J. (2007). The lipid composition of the unicellular green alga *Chlamydomonas reinhardtii* and the diatom *Cyclotella menghiniana* investigated by MALDI-TOF MS and TLC. *Chem. Phys. Lipids* 150 143–155. 10.1016/j.chemphyslip.2007.06.224 17681288

[B193] Wang K. Tu W. Liu C. Rao Y. Gao Z. Yang C. (2017). 9-cis-neoxanthin in light harvesting complexes of photosystem II regulates the binding of violaxanthin and the xanthophyll cycle. *Plant Physiol.* 174 86–96. 10.1104/pp.17.00029 28320865 PMC5411151

[B194] Wang S. Uddin M. I. Tanaka K. Yin L. Shi Z. Qi Y. (2014). Maintenance of chloroplast structure and function by overexpression of the OsMGD gene leads to enhanced salt tolerance in tobacco. *Plant Physiol.* 165 1144–1155. 10.1104/pp.114.238899 24843077 PMC4081328

[B195] Wang W. Yu L.-J. Xu C. Tomizaki T. Zhao S. Umena Y. (2019). Structrual basis for blue-green light harvesting and energy dissipation in diatoms. *Science* 363:eaav0365 10.1126/science.aav0365 30733387

[B196] Wehner A. Grasses T. Jahns P. (2006). De-epoxidation of violaxanthin in the minor antenna proteins of photosystem II, LHCB4, LHCB5, and LHCB6. *J. Biol. Chem.* 281 21924–21933. 10.1074/jbc.m602915200 16754673

[B197] Wehner A. Storf S. Jahns P. Schmid V. H. (2004). De-epoxidation of violaxanthin in light-harvesting complex I proteins. *J. Biol. Chem.* 279 26823–26829. 10.1074/jbc.m402399200 15070896

[B198] Welc R. Luchowski R. Grudzinski W. Puzio M. Sowinski K. Gruszecki W. I. (2016). A key role of xanthophylls that are not embedded in proteins in regulation of the photosynthetic antenna function in plants, revealed by monomolecular layer studies. *J. Phys. Chem. B* 120 13056–13064. 10.1021/acs.jpcb.6b10393 27976589

[B199] Williams W. P. Brain A. P. R. Dominy P. J. (1992). Induction of non-bilayer lipid phase separations in chloroplast thylakoid membranes by compatible co-solutes and its relation to the thermal stability of Photosystem II. *Biochim. Biophys. Acta* 1099 137–144. 10.1016/0304-4173(92)90019-b

[B200] Xu P. Tian L. Kloz M. Croce R. (2015). Molecular insights into zeaxanthin-dependent quenching in higher plants. *Sci. Rep.* 5:13679. 10.1038/srep13679 PMC4555179 26323786

[B201] Yamamoto H. Nakayama T. Chichester C. (1962). Studies on the light and dark interconversions of leaf xanthophylls. *Arch. Biochem. Biophys.* 97 168–173. 10.1016/0003-9861(62)90060-7 14008833

[B202] Yamamoto H. Y. (2006). Functional roles of the major chloroplast lipids in the violaxanthin cycle. *Planta* 224 719–724. 10.1007/s00425-006-0257-5 16532316

[B203] Yamamoto H. Y. Chenchin E. E. Yamada D. K. (1974). “Effect of chloroplast lipids on violaxanthin de-epoxidase activity,” in *Proceedings of the Third International Congress on Photosynthesis*, ed. Avron M. (Amsterdam: Elsevier), 1999–2006.

[B204] Yamamoto H. Y. Higashi R. M. (1978). Violaxanthin de-epoxidase. Lipid composition and substrate specificity. *Arch. Biochem. Biophys.* 190 514–522. 102251 10.1016/0003-9861(78)90305-3

[B205] Yamamoto Y. (2016). Quality control of Photosystem II: The mechanisms for avoidance and tolerance of light and heat stresses are closely linked to membrane fluidity of the thylakoids. *Front. Plant Sci.* 7:1136 10.3389/fpls.2016.01136 PMC4969305 27532009

[B206] Yamashita Y. (2018). “Recent dispersion technology using liquid crystal,” in *Liquid Crystals-Recent Advancements in Fundamental and Device Technologies*, ed. Choudhury P. K. (London: IntechOpen), 10.5772/intechopen.74156

[B207] Yan X. Chen D. Xu J. Zhou C. (2011). Profiles of photosynthetic glycerolipids in three strains of *Skeletonema* determined by UPLC-Q-TOF-MS. *J. Appl. Phycol.* 23 271–282. 10.1007/s10811-010-9553-3

[B208] Yongmanitchai W. Ward O. P. (1993). Positional distribution of fatty acids, and molecular species of polar lipids, in the diatom *Phaeodactylum tricornutum*. *J. Gen. Microbiol.* 139 465–472. 10.1099/00221287-139-3-465 20050416

[B209] Yu B. Xu C. Benning C. (2002). Arabidopsis disrupted in SQD2 encoding sulfolipid synthase is impaired in phosphate-limited growth. *Proc. Natl. Acad. Sci. U.S.A.* 99 5732–5737. 10.1073/pnas.082696499 11960029 PMC122840

[B210] Zhou F. Liu S. Yang C. (2009). Effect of monogalactosyldiacylglycerol on the interaction between photosystem II core complex and its antenna complexes in liposomes of thylakoid lipids. *Photosynth. Res.* 99 185–193. 10.1007/s11120-008-9388-9 19031112

[B211] Zhou J. Sekatskii S. Welc R. Dietler G. Gruszecki W. I. (2020). The role of xanthophylls in the supramolecular organization of the photosynthetic complex LHCII in lipid membranes studied by high-resolution imaging and nanospectroscopy. *Biochim. Biophys. Acta* 1861 148117 10.1016/j.bbabio.2019.148117 31734197

[B212] Zhu J. Gomez S. M. Mawson B. T. Jin X. Zeiger E. (1997). The coleoptile chloroplast: distinct distribution of xanthophyll cycle pigments, and enrichment in photosystem II. *Photosynth. Res.* 51 137–147.

[B213] Zhu S. H. Green B. R. (2010). Photoprotection in the diatom *Thalassiosira pseudonana*: role of LI818-like proteins in response to high light stress. *Biochim. Biophys. Acta* 1797 1449–1457. 10.1016/j.bbabio.2010.04.003 20388491

